# Physiological and genetic analysis of CO_2_-induced breakdown of self-incompatibility in *Brassica rapa*

**DOI:** 10.1093/jxb/ert438

**Published:** 2013-12-27

**Authors:** Xintian Lao, Keita Suwabe, Satoshi Niikura, Mitsuru Kakita, Megumi Iwano, Seiji Takayama

**Affiliations:** ^1^Graduate School of Biological Sciences, Nara Institute of Science and Technology, Ikoma, Nara 630-0192, Japan; ^2^Graduate School of Bioresources, Mie University, Tsu 514-8507, Japan; ^3^Tohoku Seed Co. Ltd, Utsunomiya 321-3232, Japan

**Keywords:** *Brassica rapa*, calcium, CO2, F1 hybrid, QTL, self-incompatibility.

## Abstract

Self-incompatibility (SI) of the Brassicaceae family can be overcome by CO_2_ gas treatment. This method has been used for decades as an effective means to obtain a large amount of inbred seeds which can then be used for F_1_ hybrid seed production; however, the molecular mechanism by which CO_2_ alters the SI pathway has not been elucidated. In this study, to obtain new insights into the mechanism of CO_2_-induced SI breakdown, the focus was on two inbred lines of *Brassica rapa* (syn. *campestris*) with different CO_2_ sensitivity. Physiological examination using X-ray microanalysis suggested that SI breakdown in the CO_2_-sensitive line was accompanied by a significant accumulation of calcium at the pollen–stigma interface. Pre-treatment of pollen or pistil with CO_2_ gas before pollination showed no effect on the SI reaction, suggesting that some physiological process after pollination is necessary for SI to be overcome. Genetic analyses using F_1_ progeny of a CO_2_-sensitive×CO_2_-insensitive cross suggested that CO_2_ sensitivity is a semi-dominant trait in these lines. Analysis of F_2_ progeny suggested that CO_2_ sensitivity could be a quantitative trait, which is controlled by more than one gene. Quantitative trait locus (QTL) analyses identified two major loci, *BrSIO1* and *BrSIO2*, which work additively in overcoming SI during CO_2_ treatment. No QTL was detected at the loci previously shown to affect SI stability, suggesting that CO_2_ sensitivity is determined by novel genes. The QTL data presented here should be useful for determining the responsible genes, and for the marker-assisted selection of desirable parental lines with stable but CO_2_-sensitive SI in F_1_ hybrid breeding.

## Introduction

Self-incompatibility (SI) is a widespread genetic system in many flowering plants which serves to prevent self-fertilization and maintain genetic diversity. It is based on self/non-self pollen–pistil recognition interactions followed by inhibition of self-pollen hydration, germination, or pollen tube growth.

In the Brassicaceae, SI is sporophytically controlled by a multiallelic locus termed the *S* locus (Bateman, 1955). Male and female determinants have been identified as SP11/SCR (*S*-locus protein 11/*S*-locus cysteine-rich) (Schopfer *et al.*, 1999; Takayama *et al.*, 2000) and SRK (*S* receptor kinase) (Takasaki *et al.*, 2000), respectively. When a compatible pollen grain lands on the stigma, it swells and a pollen tube is allowed to grow, whereas when self-pollen attaches to the stigma, SP11/SCR binds specifically to the extracellular domain of SRK of the same *S*-haplotype (Takayama *et al.*, 2001), which triggers an SI signalling pathway to reject self-pollen. Another stigmatically expressed gene located at the *S* locus, *S* locus glycoprotein (*SLG*) (Nasrallah *et al.*, 1987; Takayama *et al.*, 1987), has been shown to enhance the recognition process between self-pollen and stigma (Takasaki *et al.*, 2000); however, this function of *SLG* remains controversial (Silva *et al.*, 2001). A recent study suggested that the plants in the Brassicaceae genus Levenworthia use paralogous *SRK* and *SP11/SCR* genes, *Lal2* (*Leavenworthia alabamica SRK-related 2*) and *SCRl* (*SCR-like*), for self/non-self recognition in SI, but the function of their orthologues in other Brassicaceae genera also remains unknown (Chantha *et al.*, 2013).

Many studies focusing on the downstream components involved in this type of SI signalling pathway have been performed and, thus far, two components have been identified as positive effectors. ARC1 (arm repeat containing 1) was identified by a yeast two-hybrid screen using the kinase domain of SRK as the bait (Gu *et al.*, 1998; Stone *et al.*, 1999). ARC1 is a U-box protein with E3 ubiquitin ligase activity (Stone *et al.*, 2003), and has been shown to interact with Exo70A1, a putative component of the exocyst complex required for compatible pollination (Samuel *et al.*, 2009). MLPK (*M*-locus protein kinase) was identified by positional cloning as the gene responsible for the self-compatible mutation of *Brassica rapa* var. Yellow sarson (Murase *et al.*, 2004). MLPK is a membrane-anchored cytoplasmic protein kinase and interacts directly with SRK to transduce SI signalling (Kakita *et al.*, 2007). However, the importance of these two components in the Brassicaceae SI mechanism remains controversial (Kitashiba *et al.*, 2011; Indriolo *et al.*, 2012).

In the Brassicaceae, it has been known that SI can be overcome under some physiological and environmental conditions such as plant age (Ockendon, 1978; Horisaki and Niikura, 2008), stigmatic chemical treatments (e.g. ether, KOH, and NaCl) (Tatebe, 1968; Tao and Yong, 1986; Monterio and Gabelman, 1988), and high temperature (Matsubara, 1980; Okazaki and Hinata, 1987). CO_2_ gas (3–5%) treatment (Nakanishi *et al.*, 1969) is the most effective way to overcome SI. Today, most cultivated lines of crucifer vegetables, such as cabbage, broccoli, Chinese cabbage, and radish are F_1_ hybrids whose seeds are produced by mix-planting two self-incompatible inbred parental lines. In this economical F_1_ hybrid breeding system, CO_2_ gas treatment has been used to suppress SI and allow self-fertilization, thereby providing large-scale seed propagation of parental lines. This method has been used all over the world for many years, but the molecular mechanism leading to SI breakdown by CO_2_ gas treatment is entirely unknown.

Previous studies suggested that not all lines respond equally to CO_2_, and there are variations in SI response to CO_2_ (CO_2_ sensitivity) in the Brassicaceae (Nakanishi and Hinata, 1973; Niikura and Matsuura, 2000). Preliminary genetic analysis using lines with different CO_2_ sensitivity in radish (*Raphanus sativus*) suggested that high CO_2_ sensitivity was controlled by a recessive gene independent of the *S*-locus (Niikura and Matsuura, 2000). In another genetic analysis using CO_2_-sensitive and CO_2_-insensitive lines of Chinese cabbage (*B. rapa*), high CO_2_ sensitivity was suggested to be controlled by a dominant gene (Hyun *et al.*, 2007); however, no responsible genes have been identified from these studies so far.

In this study, new inbred lines of *B. rapa* with different CO_2_ sensitivity, a CO_2_-sensitive line (HA-11621) and a CO_2_-insensitive line (HA-11623), were selected and analysed. X-ray microanalysis suggested that SI breakdown in the CO_2_-sensitive line was accompanied by significant calcium accumulation at the pollen–stigma interface. Independent pre-treatment of pollen or pistil with CO_2_ gas before pollination showed no effect on the SI reaction, suggesting that some physiological process that occurs after pollination is necessary for SI to be overcome. Genetic analyses using F_1_ and F_2_ progeny of a CO_2_-sensitive×CO_2_-insensitive cross suggested that CO_2_ sensitivity is a semi-dominant and quantitative trait. Furthermore, quantitative trait locus (QTL) analyses identified two major responsible loci, *BrSIO1* and *BrSIO2*, which function additively in overcoming SI during CO_2_ treatment.

## Materials and methods

### Plant materials

Two inbred lines of *B. rapa* (2*n*=20), a CO_2_-sensitive line (HA-11621) and a CO_2_-insensitive line (HA-11623), were established at Tohoku Seed Co., Ltd, and grown in the greenhouse with 16h light and 8h dark conditions at 20 °C. Both lines show stable SI under normal (open-air) condition but have different CO_2_ sensitivity; SI in HA-11621 breaks down following treatment with 4.5% CO_2_ whereas SI in HA-11623 is unaffected. HA-11621 and HA-11623 are reciprocally compatible, and their F_1_ progeny were obtained under normal conditions. Buds (1–2 d before flowering) from a randomly chosen F_1_ were used for F_2_ production. Young petals and stamens were removed from the bud, and the immature pistil was pollinated with pollen grains from mature flowers of the same plant (bud pollination). Pollinated pistil was then covered with a paper bag for 3 d and seeds from the pistil were harvested. More than 20 pistils were pollinated, and harvested seeds were used as the F_2_ population. A total of 110 F_2_ plants were used for phenotypic and genetic analysis.

### Cryo-scanning electron microscopy and energy-dispersive X-ray analysis

Flowers were self- or cross-pollinated and incubated for 1.5h with or without 4.5% CO_2_ gas. These pollinated and non-pollinated pistils were submersed in liquid nitrogen slush and frozen under vacuum. While under vacuum, the sample was transferred to the microscope cryo stage (ALTO 1000, Gatan), and then the stage temperature was increased to –95 °C to remove frost that had settled on top of the specimen as a result of condensation. When all surface frost had been removed by sublimation, as verified by electron microscopy, the temperature was reduced to –140 °C. Imaging was performed using an ETD (Everhart–Thornley detector) by Quant 250 scanning electron microscopy (FEI). The chamber pressure was 30 Pa and the accelerating voltage was 15kV. EDX (energy-dispersive X-ray spectroscopy) analysis of the element assay was performed on selected papilla cells using INCA X-ray analysis software (Oxford Instruments, http://www.oxinst.com/Pages/home.aspx, last accessed 14 December 2013), with the detector’s processing time set at 2. X-ray data were collected with 4.5 nA probe current for 2min. Each 2–3 pistils were used in one experiment and three individual experimental sets were performed.

### Evaluation of reaction level of SI to CO_2_ (RLSICO_2_)

Three to five flowers were cut at the peduncle and stood on a 1% (w/v) solid agar plate. Flowers were self-pollinated, placed into a CO_2_ incubator, and treated with 4.5% CO_2_ for 4h at 23 °C. After 1 d at room temperature, pistils were fixed in ethanol:acetic acid (3:1) overnight, softened in 1 N NaOH at 60 °C for 2h, then stained with 0.01% (w/v) decolorized aniline blue in 2% K_3_PO_4_ for 6h. Pollen tube behaviour was observed under a fluorescent microscope (Axiophot 2, Zeiss). CO_2_ sensitivity was measured using the RLSICO_2_ index. RLSICO_2_ was classified into five categories, based on the number of pollen tubes penetrating into the stigma: 1, 0 pollen tubes; 2, 1–5 pollen tubes; 3, 6–15 pollen tubes; 4, 16–30 pollen tubes; and 5, >30 pollen tubes. Three replicates were performed on each plant on different days. Non-CO_2_-treated self-pollinated flowers were used as controls. In all cases, no pollen tubes penetrated into the control stigmas.

### Genotyping of *S*-haplotypes

*S*-haplotypes of *B. rapa* were identified using primers PS5 (5′-ATGAAAGGCGTAAGAAAAACCTA-3′) and PS15 (5′-CCG TGTTTTATTTTAAGAGAAAGAGCT-3′) (Nishio *et al.*, 1996) to amplify a fragment of the *SLG* gene. PCR-RFLP (restriction fragment length polymorphism) was used to distinguish the two *S*-haplotypes based on differential digest with the restriction enzyme *Kpn*I (TaKaRa, Japan). Digested DNA was electrophoresed on a 1.5% agarose gel.

### Molecular markers and detection of DNA polymorphism

To screen for markers that show polymorphism between *B. rapa* lines, primers specific for simple sequence repeat (SSR) markers from different sources [UK, prefixes Ra, Na, Ol, and ENA (Lowe *et al.*, 2004; http://brassica.bbsrc.ac.uk); Japan, prefixes BRMS, KBr, and EST (Suwabe et al., 2002, 2004, 2006; http://vegmarks.nivot.affrc.go.jp, NIVTS); China, prefix sau_um (Ge *et al.*, 2011); and Korea, prefix AMCP (Ramchiary *et al.*, 2011)] were used. SSR, RFLP, and insertion/deletion (InDel) markers (prefixes XT and Bra) were also designed based on the *Brassica* database (BRAD) (http://brassicadb.org/brad/, last accessed 14 December 2013) (Supplementary Table S1 available at *JXB* online).

Total genomic DNA was extracted from young leaves of two parental lines and F_2_ progeny using the cetyltrimethylammonium bromide (CTAB) method (Murray and Thompson, 1980). DNA polymorphism analysis with SSRs was carried out using PCR with fluorescent dyes, performed according to Suwabe *et al.* (2008) with some modifications. The M13 (–21) universal primer sequence (18bp) was fused to the 5′ end of the original forward primer, and the M13 (–21) universal primer was labelled with 6-FAM, NED, VIC, or PET fluorescent dye (Applied Biosystems, CA, USA). PCRs were performed in a 10 μl reaction volume containing 10ng of template DNA, 4.7 μM of labelled M13 (–21) universal primer and reverse primer, 0.3 μM of forward primer, 1× PCR buffer, 1× dNTP, 1× MgCl_2_, and 0.5U of rTaq (TOYOBO, Japan). Conditions for PCR were as follows: initial denaturation was carried out at 94 °C for 3min followed by 37 cycles at 94 °C for 30 s, 55 °C (slope of 0.5 °C s^–1^) for 30 s, 72 °C (slope of 0.5 °C s^–1^) for 30 s, and a final extension at 72 °C for 4min. A 1 μl aliquot of 50-fold diluted PCR product was added to 8.9 μl of Hi-Di™ Formamide and 0.1 μl of GeneScan™ 600 LIZ™ Size Standard (Applied Biosystems, USA) and applied to an ABI 3730 DNA Analyzer (Applied Biosystems). Data were analysed using ABI GeneMapper^®^ software.

For polymorphism analysis with RFLP and InDel markers, PCR was carried out in a 10 μl reaction volume with 5 pmol of forward and reverse primers instead of fluorescent dyes. For RFLP markers, amplified fragments were digested using restriction enzymes for 1h. Fragments of digested DNA were separated on a 2–4% agarose gel.

### Linkage map construction and QTL analysis

A genetic map was constructed using JoinMap^®^ version 4 (Van Ooijen, 2006) utilizing the double pseudo-testcross strategy with a log_10_ of odds (LOD) threshold of 6.0 for linkage group identification. The best marker order was calculated with the regression mapping algorithm, and marker order was retained from the first round only. Map distance units in centiMorgans (cM) were converted from recombination frequencies using the Kosambi mapping function (Kosambi, 1944). Interval mapping (IM) was performed to identify putative QTLs using the established linkage map and the observed phenotypic traits. This method was run using MapQTL^®^ version 6 (Van Ooijen, 2009). With this software, a *P* < 0.05 LOD score significance threshold was calculated by creating a group-wide distribution of the data based on a 1000 permutation test. LOD peaks were used to estimate the position of QTLs on the map.

### Statistical analysis

Box plots were prepared by Ekuseru-Toukei 2012 software (Social Survey Research Information Co., Ltd, Japan) to compare the phenotypic difference, as this plot type gives a good sense of environmental data distribution (Upton and Cook, 1996). Kruskal–Wallis analysis of variance (ANOVA) by ranks was used between paired comparisons of markers to examine marker association.

## Results

### Phenotypic analysis of *B. rapa* lines in response to CO_2_ treatment

**Fig. 1.**
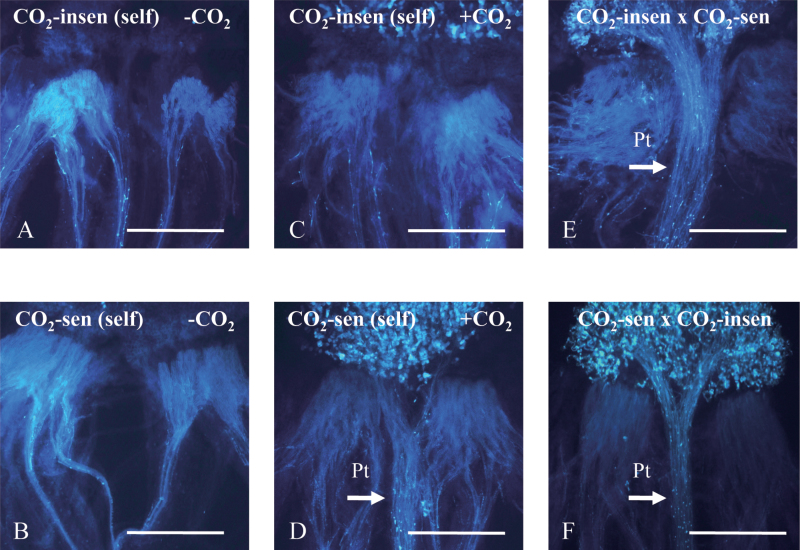
Phenotype of inbred *Brassica rapa* lines used in this study. (A, B) Pollen tube behaviour after self-pollination of CO_2_-sensitive (HA-11621) and CO_2_-insensitive (HA-11623) lines under normal conditions (without CO_2_ treatment). No penetrated or elongated pollen tubes are observed in either line. (C, D) Pollen tube behaviour after self-pollination of CO_2_-sensitive and CO_2_-insensitive lines under 4.5% CO_2_ gas treatment. Pollen tubes could penetrate into the stigma and elongate through the style only in the CO_2_-sensitive line. (E, F) Cross-pollination as a positive control; the arrow shows pollen tubes which have penetrated. Pt, pollen tubes. Bar=1000 μm.

Two inbred lines of *B. rapa* with different CO_2_ sensitivity, a CO_2_-sensitive line (HA-11621) and a CO_2_-insensitive line (HA-11623), were used in this study. Flowers were self-pollinated by hand pollination and incubated in a CO_2_ incubator (4.5% CO_2_) for 4h. Both lines were self-incompatible under normal conditions (control) (Fig. 1A, B), whereas they showed significantly different responses to CO_2_ gas treatment (Fig. 1C, D). Specifically, in the CO_2_-sensitive line, many pollen tubes were seen to penetrate into papilla cells after CO_2_ treatment. This pollination test confirmed that the CO_2_-sensitive line had high sensitivity to CO_2_ while the CO_2_-insensitive line hardly responded to 4.5% CO_2_. Cross-pollination was performed as a positive control (Fig. 1E, F).

### Physiological changes in papilla cells after CO_2_ treatment

**Fig. 2.**
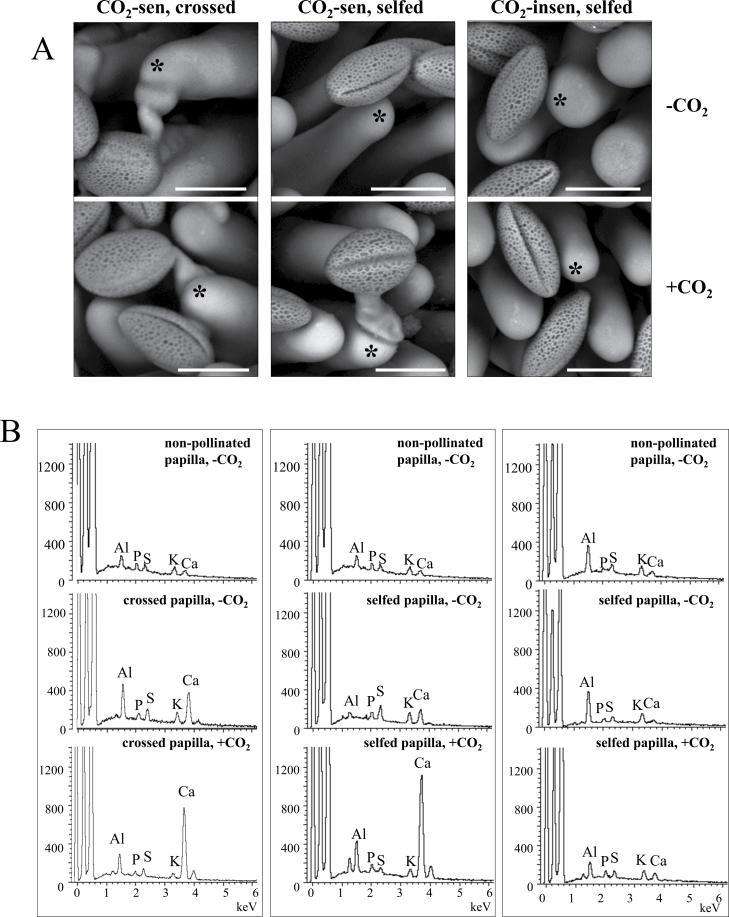
Electron micrographs and X-ray microanalysis of *B. rapa*. (A) Cryo-scanning electron micrographs of pollinated papilla cells were taken 1.5h after pollination. Representative examples of the cross-pollinated (left column) and self-pollinated (middle column) CO_2_-sensitive line, and the self-pollinated (right column) CO_2_-insensitive line are shown. Without CO_2_ treatment (upper panels), only cross-pollen is accepted, and self-pollen grains maintain a spheroid shape without swelling in both lines. With 4.5% CO_2_ gas treatment (lower panels), pollen grains swell and germinate in the CO_2_-sensitive line (lower middle) but not in the CO_2_-insensitive line (lower right). Bar=25 μm. (B) Representative examples of energy-dispersive X-ray spectra of non-pollinated and pollinated papilla cell surfaces. Scanning positions for X-ray analyses are indicated by asterisks in (A). The emissions of Al-Kα, P-Kα, S-Kα, K-Kα, and Ca-Kα were detected at the papilla cell surfaces, and the intensity of Ca emission was increased after cross-pollination (left column). The increase of Ca emission was also observed after self-pollination with 4.5% CO_2_ gas treatment in the CO_2_-sensitive line (middle column) but not in the CO_2_-insensitive line (right column). These spectrum patterns are reproducible in three individual experiment sets. The emission of Al-Kα is mostly derived from the stub that held the samples.

Previous work using X-ray microanalysis has revealed the accumulation of calcium at the stigmatic surface following compatible cross-pollination in *Brassica oleracea* (Elleman and Dickinson, 1999). X-ray mapping strongly indicated that a high concentration of calcium was localized at the points where the pollen grain made contact with the surface of the stigmatic papilla cell. The calcium accumulation was also observed in *B. rapa*, especially in compatible pollination (Iwano *et al.*, 1999). To examine the physiological effect of CO_2_ on pollination reactions, this calcium accumulation was examined by using cryo-scanning electron microscopy fitted with an X-ray microanalysis system. When the CO_2_-sensitive line was cross-pollinated with the CO_2_-insensitive line, pollen grain hydrated and the pollen tube penetrated into the papilla cell, but when CO_2_-sensitive and CO_2_-insensitive lines were self-pollinated under normal conditions (without CO_2_ treatment), few pollen grains hydrated, and no pollen tube germination was observed in either line (Fig. 2A, upper panel). After CO_2_ treatment, the cross-pollinated pollen grains did not show a significant difference, neither did the self-pollinated CO_2_-insensitive line. However, the self-pollinated CO_2_-sensitive line showed obvious changes: pollen hydration and germination were observed under CO_2_ treatment (Fig. 2A, lower panel). The emission of elements (Kα) in the tip of the papilla cells was then analysed after selecting the pollinated papilla cells which faced nearly the same direction in relation to the X-ray detector. Emissions of P-Kα, S-Kα, K-Kα, and Ca-Kα were detected together with C and O, which are constituent elements of biological materials. The detected emission of Al-Kα originates from the stub that held the samples (Fig. 2B). There was no significant difference in the elemental emissions between CO_2_-sensitive and CO_2_-insensitive lines before pollination. Ca^2+^ accumulation was observed after cross-pollination as previously reported (Iwano *et al.*, 1999), and Ca-Kα emission was increased with CO_2_ treatment. After self-pollination, Ca-Kα emission was slightly increased in the CO_2_-sensitive line in the normal CO_2_ condition. This Ca-Kα increase became more pronounced (~6-fold) in the CO_2_-sensitive line when it was self-pollinated in the high CO_2_ condition. In the CO_2_-insensitive line, no significant Ca-Kα increase was observed after self-pollination in normal or high CO_2_ conditions (Fig. 2B). Although the biological significance of the accumulation of calcium at the pollen–stigma interface is not clear, it has been suggested that calcium plays some role in the successful development of the pollen tube tip into the region of expanded stigmatic wall (Elleman and Dickinson, 1999). The present results suggest that, in the CO_2_-sensitive line, high CO_2_ activates a compatible pollination pathway or disturbs an SI signalling pathway leading to Ca^2+^ accumulation at the pollen–stigma interface.

### The efficiency of CO_2_ treatment

**Fig. 3.**
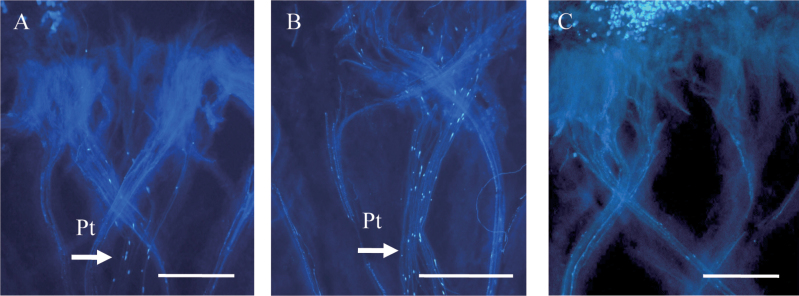
The efficiency of CO_2_ treatment of the CO_2_-sensitive line. (A) Self-pollinated stigmas were treated with 4.5% CO_2_ immediately after self-pollination, and pollen tube penetration and elongation were observed. (B) Self-pollinated stigmas were treated with 4.5% CO_2_ 3h after self-pollination. The pollen tube can still penetrate into papilla cells. (C) When CO_2_ treatment began 6h after self-pollination, the number of penetrating pollen tubes was decreased. Pt, pollen tubes. Bar=1000 μm.

A previous study suggested that the effect of CO_2_ on SI breakdown depends on the timing of treatment (Nakanishi and Hinata, 1973). In the CO_2_-sensitive line, when self-pollinated flowers were immediately treated with 4.5% CO_2_ for 4h, SI could be overcome, and typically >10 pollen tubes penetrated into the stigma (Fig. 3A). When CO_2_ treatment started 3h after self-pollination, SI could still be overcome (Fig. 3B). However, when CO_2_ treatment started 6h after self-pollination, the number of penetrating pollen tubes was decreased (Fig. 3C). These results indicate that self-pollen inhibition in SI is biostatic, as previously suggested (Sarker *et al.*, 1988), and can be reversed at least at 3h after pollination. However, at 6h after pollination, SI inhibition enters an irreversible phase that cannot be overcome by CO_2_ treatment.

**Fig. 4.**
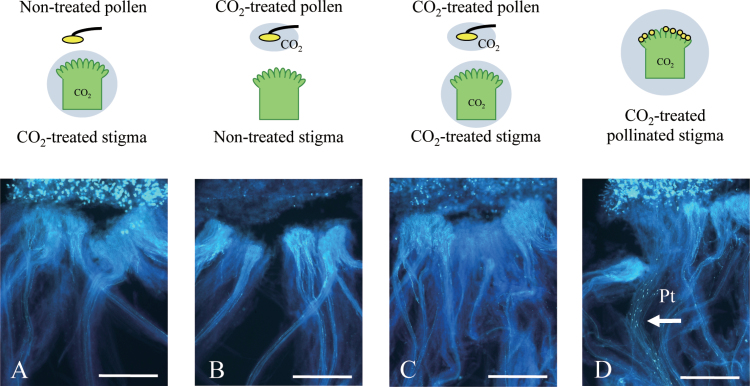
Pollination assay using CO_2_-treated non-pollinated flowers of the CO_2_-sensitive line as either CO_2_-treated pistil or pollen. Pollen tube behaviour is observed after a 4h CO_2_ treatment immediately after self-pollination. (A) A pistil from a non-pollinated flower treated with CO_2_ for 4h was pollinated with pollen from the same plant which was not treated with CO_2_. (B) A pistil from a non-treated non-pollinated flower was pollinated with pollen from the same plant which was treated with CO_2_ for 4h. (C) A pistil from a CO_2_-treated non-pollinated flower (CO_2_-treated) was pollinated with pollen from the same plant which was treated with CO_2_ for 4h. (D) Self-pollinated flower treated with CO_2_ after pollination, positive control. Pt, pollen tubes. Bar=1000 μm.

Next, in order to narrow down the stage of SI affected by CO_2_ treatment, experiments with high CO_2_-pre-treated pollen or pistil from the CO_2_-sensitive line were performed (Fig. 4). SI could not be overcome by pre-treatment of either tissue, and no pollen tube penetration could be observed even when both pollen and pistil were treated separately prior to pollination (Fig. 4). SI could be overcome only when the self-pollinated pistil was treated with high CO_2_. These results suggest that some post-pollination physiological process is affected by high CO_2_, in the process of SI breakdown.

### *S*-allele characterization and phenotype of CO_2_ sensitivity in F_1_ and F_2_

**Fig. 5.**
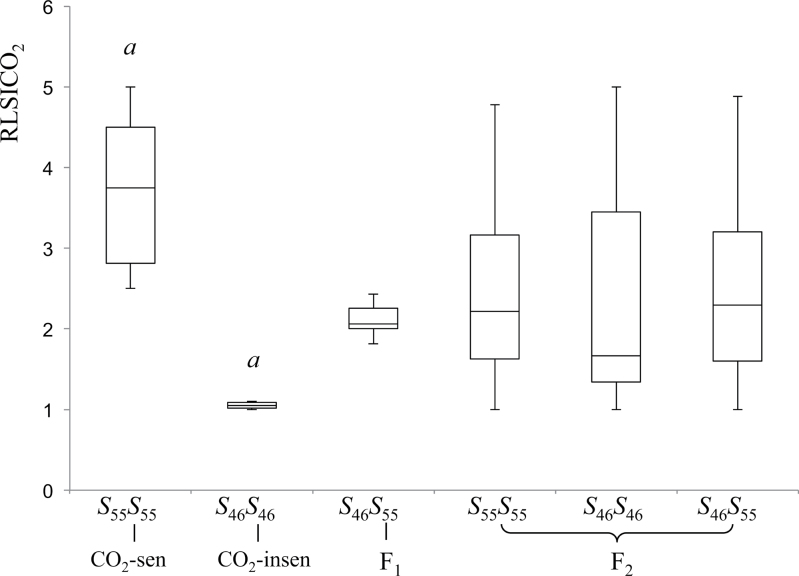
Box plots of CO_2_ sensitivity phenotypes. Data show the distribution of RLSICO_2_ with 25th, 50th, and 75th percentiles (horizontal bars), interquartile ranges (columns), and 1.5 interquartile ranges (error bars) of RLSICO_2_ from six CO_2_-sensitive (*S*_55_ *S*_55_) and six CO_2_-insensitive (*S*_46_ *S*_46_), individuals, six F_1_ individuals (*S*_46_ *S*_55_), and 110 F_2_ individuals (22 *S*_46_ *S*_46_, 64 *S*_46_ *S*_55_, and 24 *S*_55_ *S*_55_). *a* indicates a significant difference (*P* < 0.01) between CO_2_-sensitive and CO_2_-insensitive lines.

The *S*-haplotypes of the two parental inbred lines were first determined by amplifying their *SLG* genes (Nishio *et al.*, 1996). The sequence data suggested that the *S*-haplotypes of the CO_2_-sensitive and CO_2_-insensitive lines were *S*_55_ *S*_55_ and *S*_46_ *S*_46_, respectively. To dissect genetically the gene(s) that determines sensitivity to CO_2_ treatment, six F_1_ plants (*S*_46_ *S*_55_) were produced by crossing CO_2_-sensitive and CO_2_-insensitive lines. These F_1_ plants exhibited an intermediate CO_2_ sensitivity phenotype where self-pollen tubes penetrated into the stigma under high CO_2_ treatment but there were fewer penetrating pollen tubes than observed in a self-pollination of the CO_2_-sensitive parent. An F_2_ population of 110 individuals derived from a bud-pollinated F_1_ plant was made and used for further genetic analyses of the CO_2_ sensitivity. F_2_ individuals were genotyped using PCR-RFLP to distinguish *SLG* alleles (Supplementary Fig. S1 at *JXB* online). *S*_55_- and *S*_46_-haplotypes were segregated in the F_2_ population according to Mendelian transmission (Supplementary Table S2). Pollen tube behaviour after CO_2_ treatment varied among individuals and, in order to quantify the strength of CO_2_ sensitivity, the modified RLSICO_2_ (reaction level of SI to CO_2_) index was employed (Niikura and Matsuura, 2000), which calculates CO_2_ sensitivity based on the number of penetrating pollen tubes after self-pollination under high CO_2_ conditions (see the Materials and methods). The RLSICO_2_ of 110 F_2_ individuals is presented in Supplementary Fig. S2, and the summarized box-plot data are shown in Fig. 5, together with the RLSICO_2_ of F_1_ and the parental inbred lines. F_1_ had an RLSICO_2_ score intermediate to the two parental lines, suggesting that the high CO_2_ sensitivity is a semi-dominant (incompletely dominant) trait in these inbred lines. Furthermore, the RLSICO_2_ of F_2_ individuals was continuously distributed and did not follow a simple one-locus biallelic Mendelian distribution (Supplementary Fig. S2). These results suggest that CO_2_ sensitivity in the inbred lines used here could be a quantitative trait which is controlled by more than one gene.

### Relationship between *S*-alleles and CO_2_ sensitivity

To investigate whether CO_2_ sensitivity is related to *S*-haplotypes, the 110 F_2_ individuals were grouped into three genotypes (*S*_55_ *S*_55_, *S*_46_ *S*_46_, and *S*_46_ *S*_55_). The RLSICO_2_ of each group is shown in Fig. 5. In the three F_2_ groups, RLSICO_2_ scores were distributed from 1 to 5, and interquartile ranges overlapped, indicating that CO_2_ sensitivity is not linked to the *S*-locus in these two lines.

### Reproductive tissue controlling CO_2_ sensitivity

**Fig. 6.**
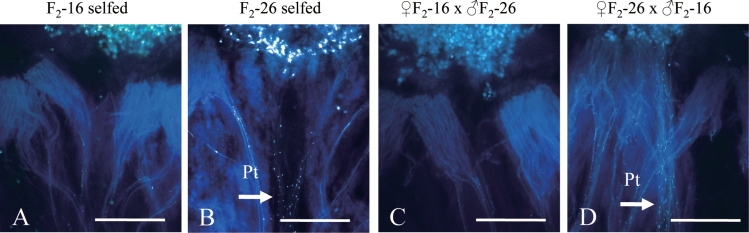
Reciprocal crosses with CO_2_ treatment between two *S*_46_ homozygous individuals from the F_2_ population with different RLSICO_2_. (A) CO_2_-sensitive F_2_ self-pollination. (B) CO_2_-insensitive F_2_ self-pollination. (C) A CO_2_-sensitive F_2_ pistil pollinated with pollen from a CO_2_-insensitive F_2_. (D) A CO_2_-insensitive F_2_ pistil pollinated with pollen from a CO_2_-sensitive F_2_. Pt, pollen tubes. Bar=1000 μm. *n*=3.

From the F_2_ population, two *S*_46_ *S*_46_ homozygotes with different RLSICO_2_ were selected: F_2_-16, a CO_2_-insensitive line (RLSICO_2_=1±0); and F_2_-26, a CO_2_-sensitive line (RLSICO_2_=4.15±0.53). These two lines were used to examine the reproductive tissue controlling CO_2_ sensitivity. Reciprocal crosses were performed between these two SI lines with or without high CO_2_ gas treatment. Because all crosses under normal conditions (without CO_2_ treatment) were incompatible, only data from crosses performed in the high CO_2_ condition are shown in Fig. 6. The cross between CO_2_-sensitive F_2_-26 pistil and CO_2_-insensitive F_2_-16 pollen was CO_2_ sensitive, showing many penetrating pollen tubes under high CO_2_. On the other hand, the cross between CO_2_-insensitive F_2_-16 pistil and CO_2_-sensitive F_2_-26 pollen was CO_2_ insensitive, showing no penetrating pollen tubes even under high CO_2_. These results suggest that CO_2_ sensitivity is controlled by genes expressed in the female tissue (pistil).

### Marker analysis and construction of a linkage map

**Fig. 7.**
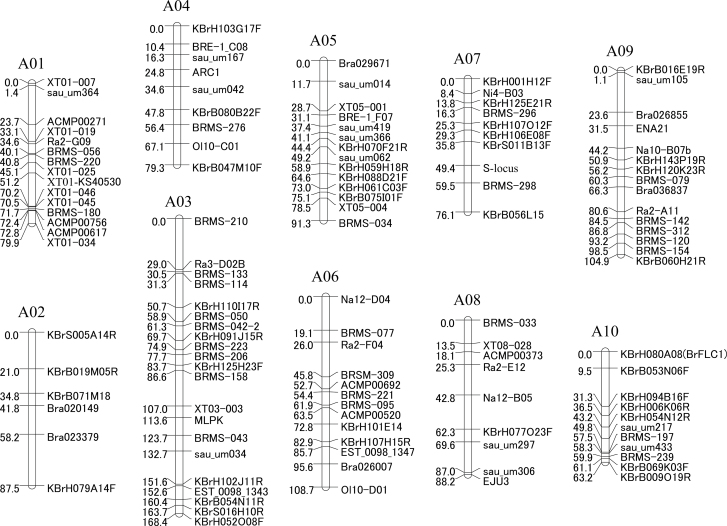
A linkage map of selected DNA markers from the F_2_ population. Map distances are shown to the left of vertical lines of the linkage group in cM, and marker names are shown to the right.

In order to map QTLs that determine CO_2_ sensitivity, a linkage map was constructed for this F_2_ population. A total of 911 different genetic markers were examined in the two parental lines. To clarify the relationship between previously identified SI-related genes and CO_2_ sensitivity, *SLG* (an *S*-locus marker), *MLPK*, and *ARC1* were also selected. Though a very low level of polymorphism (14.7%) was detected for all types of markers, 123 polymorphic markers were selected, which include 113 SSRs, five RFLPs, and five InDel markers. These 123 markers were used for linkage mapping, and generated 10 linkage groups (A01–A10) at a LOD threshold value of 6.0 (Fig. 7). The total length of the map was 947.5 cM, and the length of the linkage groups ranged from 63.2 cM (A10) to 168.4 cM (A03). The distance between markers varied from 0 to 29.3 cM, with an average interval of 7.7 cM. *SLG*, *MLPK*, and *ARC1* were mapped to A07, A03, and A04, respectively, which is consistent with previous reports (Ajisaka *et al.*, 2001; Hatakeyama *et al.*, 2010).

### QTL analysis and association of markers with high CO_2_ sensitivity

**Fig. 8.**
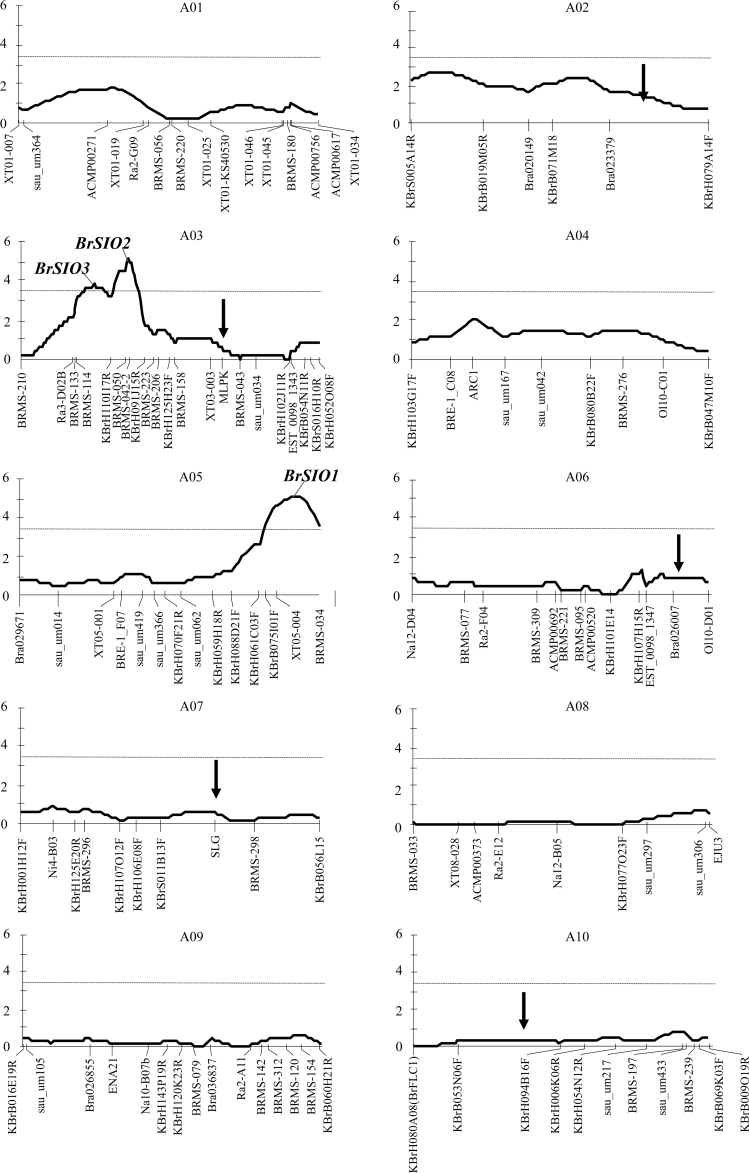
QTL analysis results. The solid line indicates the LOD score and the dotted line indicates the QTL threshold (LOD=3.4) determined using a 1000 permutation test (*P* < 0.05). The *x*-axis represents each linkage group (cM) and the *y*-axis indicates the QTL score. Two QTLs (*BrSIO2* and *3*) are detected in A03 and one in A05 (*BrSIO1*). Arrows show loci involved in SI stability reported by Hatakeyama *et al.* (2010).

Using the constructed linkage map, QTLs responsible for high CO_2_ sensitivity were analysed. Three QTLs were identified on linkage groups A03 and A05 based on a LOD threshold of 3.40 (1000 permutation test, *P* < 0.05) (Fig. 8, Table 1). These QTLs were tentatively named *Brassica rapa SI Overcome (BrSIO) 1–3*, and these results further supported the prediction that CO_2_ sensitivity of SI is controlled by a polygenic system. *BrSIO1* on A05 and *BrSIO2* on A03 are two major QTLs that explained 19.3% and 19.0% of phenotypic variation, respectively. *BrSIO3*, located near *BrSIO2*, accounted for 14.5% of the variance (Table 1).

**Table 1.** Summary of CO_2_ sensitivity QTLs

QTL	LG	Closest marker	QTL peak (cM)^*a*^	LOD	*R* ^2*b*^	Additive effect^*c*^
*BrSIO1*	A05	XT05-004	83.50	5.17	19.30	0.72
*BrSIO2*	A03	BRMS-042-2	60.87	4.46	19.00	0.69
*BrSIO3*	A03	KBrH110I17R	41.25	3.76	14.50	0.65

^*a*^ QTL peak position, detected by interval mapping, between two markers.

^*b*^ Amount of phenotypic variation explained by the QTL.

^*c*^ Additive effect of the CO_2_-sensitive HA-11621 allele on RLSICO_2_.

To examine the significance of these QTLs, F_2_ progeny were classified into groups based on the genotypes of the linkage markers nearest these three newly identified loci, and the relationship of the loci to RLSICO_2_ in individual plants was analysed using Kruskal–Wallis ANOVA by ranks (Table 2). Alleles from CO_2_-sensitive (HA-11621) and CO_2_-insensitive (HA-11623) lines are presented as *S* and *I*, respectively. Almost all classifications using the closest linkage markers showed a higher RLSICO_2_ index in the *SS* group with significance at *P* < 0.01, except marker BRMS-114, which showed significance at *P* < 0.05.

**Table 2.**
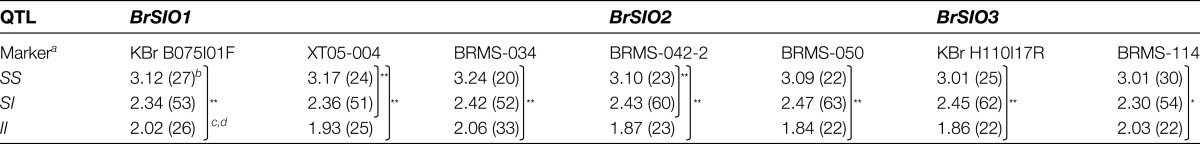
Statistical analysis of QTL effect

^*a*^ *S*, CO_2_-sensitive HA-11621 allele; *I*, CO_2_-insensitive HA-11623 allele.

^*b*^ Individuals whose genotype was unidentified are excluded.

^*c*^ Kruskal–Wallis analysis comparing phenotype between genotype groups with individuals in the same groups.

^*d*^ Significance level: **P <0.01; *P <0.05.

Marker association was further examined with combinations of *BrSIO1* and *BrSIO2* since *BrSIO3* is a minor QTL closely linked to *BrSIO2*, making it difficult to identify it as an independent QTL. F_2_ progeny were classified into nine groups based on the genotypes of their closest linkage markers (Table 3). According to this classification, for example, the two above analysed *S*_46_ *S*_46_ homozygous lines with different RLSICO_2_, F_2_-16 (CO_2_-insensitive line) and F_2_-26 (CO_2_-sensitive line), were classified into group 8 and group 1, respectively. When groups with the same *BrSIO1* genotype were compared, the *BrSIO2 SS* group showed a higher RLSICO_2_ index compared with the *BrSIO2 II* group. Likewise, when the groups with the same *BrSIO2* genotype were compared, the *BrSIO1 SS* group showed a higher RLSICO_2_ compared with the *BrSIO1 II* group. Although the numbers of F_2_ individuals in each group were rather low, significance (*P* < 0.05) was detected between groups 1 and 6, 2 and 6, and 2 and 8. These data suggest that *BrSIO1* and *BrSIO2* work additively in overcoming SI during CO_2_ treatment in the CO_2_-sensitive (HA-11621) line. No QTL was detected at genes known to affect SI stability (*MLPK*, *ARC1*, or the *S*-locus), indicating that CO_2_ sensitivity is determined by novel genes in the experimental lines used here.

**Table 3.**
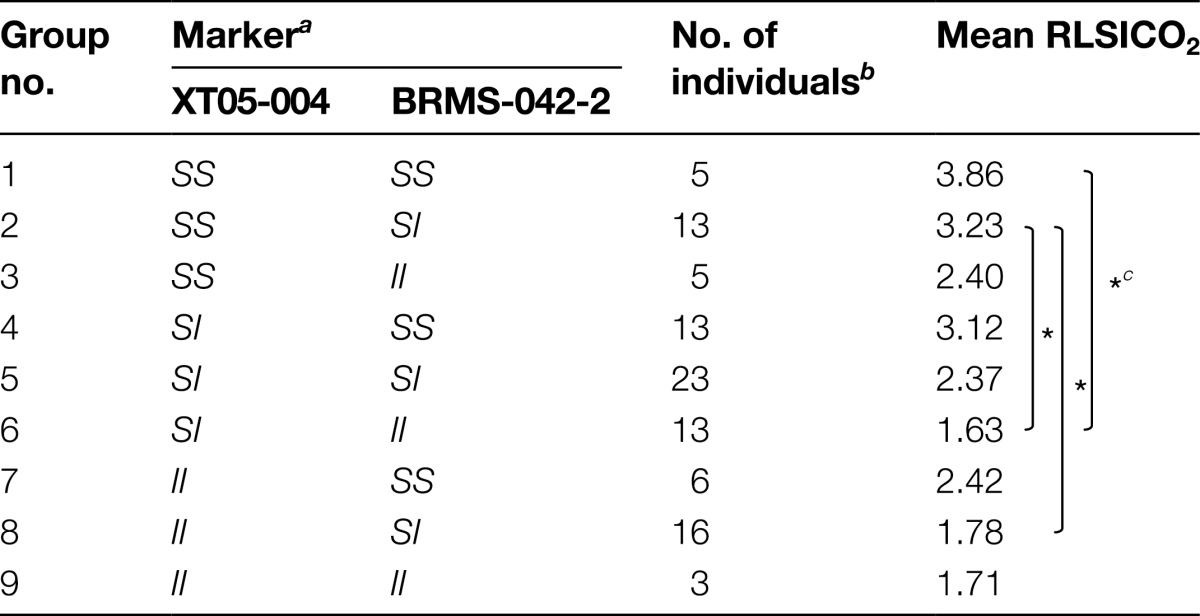
QTL association for CO_2_ sensitivity

^*a*^ *S*, CO_2_-sensitive HA-11621 allele; *I*, CO_2_-insensitive HA-11623 allele.

^*b*^ Individuals whose genotype was unidentified are excluded.

^*c*^ Significance level, *P <0.05.

### Associated gene prediction by *in silico* comparative mapping

Using the *B. rapa* genome sequence (Cheng *et al.*, 2011), *BrSIO1* could be mapped to a 569kb region flanked by InDel marker XT05-004 and SSR marker BRMS-034, and *BrSIO2* to a 1469kb region flanked by SSR markers BRMS-042-2 and KBrH110I17R. These two regions include 121 and 280 genes annotated in the *Brassica* database (BRAD), respectively (Supplementary Tables S3, S4 at *JXB* online). Comparison of the *A. thaliana* genome with the Brassicaceae genome (reviewed by Schranz *et al.*, 2006) suggests that *BrSIO1* has synteny on *A. thaliana* chromosome 2 and *BrSIO2* has synteny on both chromosomes 3 and 4. It is assumed that these two QTLs do not have the same genetic origin and could be two independent regions controlling high CO_2_ sensitivity. Based on reciprocal cross results, the CO_2_ sensitivity trait may be controlled by genes expressed in the female organ (Fig. 8). A total of 121 and 280 annotated genes in *BrSIO1* and *BrSIO2* have 103 and 243 homologues in *A. thaliana*, respectively, and 54 and 141 of these genes are expressed in *A. thaliana* pistil (microarray data of carpel at stage 12, http://affymetrix.arabidopsis.info/narrays/search.pl?f1=1&s1=ATGE_37, last accessed 14 December 2013, Supplementary Tables S3, S4).

Genes involved in related biological processes are often expressed cooperatively and their co-expression information is important for understanding biological systems (Eisen *et al.*, 1998). ATTED-II (http://atted.jp/, last accessed 14 December 2013) is a gene co-expression database useful for identifying the potential partners working in the same biological processes (Obayashi *et al.*, 2007). Co-expression analysis was performed using ATTED-II with these 195 genes and it was found that *MAP kinase 6* (At2g43790 in *BrSIO1*) and *ethylene overproducer 1* (At3g511770 in *BrSIO2*) showed the strongest co-expression and *calmodulin-like 41* (At3g50770 in *BrSIO2*) has weak co-expression with *cytochrome c oxidase 10* (At2g44520in *BrSIO1*) and *beta glucosidase 28* (At2g44460 in *BrSIO1*). In addition to these co-expressed genes, these two regions encode highly homologous family member proteins, for example matrixin proteins (At2g45040 in *BrSIO1* and At4g16640 in *BrSIO2*) and senescence-associated proteins (At2g44670 in *BrSIO1* and At4g17670 in *BrSIO2*). All these can be candidate responsible genes, although the biological functions of these genes are mostly unknown.

## Discussion

It has been >40 years since Nakanishi *et al.* (1969) first reported that SI could be overcome by CO_2_ and Nakanishi and Hinata (1973) demonstrated the applicability of this technique to commercial use. Nowadays, seed companies have adopted this method to obtain inbred parental seeds of crucifer vegetables for large-scale commercial F_1_ hybrid seed production. However, there is still very limited understanding of the mechanism by which SI is overcome.

Lee *et al.* (2001) showed a shrunken and distorted papilla cell surface in the CO_2_-sensitive *B. rapa* cv. Hiratsuka, and suggested that these structural changes could cause SI to be overcome. The cryo-scanning electron microscopy data reported here did not show any structural changes in CO_2_-sensitive or CO_2_-sensitive lines (Fig. 2A). Additionally, pre-treatment of non-pollinated pistils with high CO_2_ gas did not cause SI breakdown (Fig. 4). Therefore, a completely different SI breakdown mechanism must be present, at least in the CO_2_-sensitive line. In contrast, massive Ca accumulation was observed at the pollen–stigma interface specifically in CO_2_-sensitive plants under high CO_2_ conditions (Fig. 2B). Brewbaker and Kwack (1963) were the first to describe the need for a high concentration of Ca^2+^ for pollen germination and pollen tube growth. The high concentration of Ca^2+^ could be needed for activating pectinase to loosen the papilla cell wall, allowing the pollen tube to penetrate (Black and Charlwood, 1995), or for keeping the pollen tube cell wall rigid enough not to burst (Hepler and Winship, 2010). Although causal relationships remain unclear, the data suggest that CO_2_ treatment induces a certain compatible reaction leading to Ca^2+^ accumulation at the pollen–stigma interface.

To date, several genetic studies have been performed to understand the mechanism of SI breakdown for breeding purposes. Niikura and Matsuura (2002) reported that in Japanese radish high CO_2_ sensitivity is controlled by a recessive gene that governs the construction and/or metabolism of the stigma, which reacts to CO_2_ without any changes in gene expression. In contrast, Hyun *et al.* (2007) reported a dominant, *S*-haplotype-linked high CO_2_ sensitivity phenotype in *B. rapa*. In contrast to these previous reports, F_1_ plants had an intermediate CO_2_ sensitivity and the F_2_ population had a continuous frequency distribution of RLSICO_2_ in the present study (Fig. 6). These results suggest that in the lines used for this study, CO_2_ sensitivity is a quantitative trait which is controlled by more than one gene.

Genetic linkage maps based upon frequency of recombination in segregating populations are fundamental and powerful tools for associating phenotypic trait-specific genetic regions. Linkage mapping can be used to understand the biological basis of complex traits and to dissect genetic determinants underlying the expression of agronomically important breeding traits (Paran and Zamir, 2003). Using QTL analysis, two major QTLs for high CO_2_ sensitivity were successfully identified (Fig. 8). *BrSIO1* and *BrSIO2* had similar LOD scores and explained similar amounts of phenotypic variation (19.3% and 19%), and these could be two major factors controlling high CO_2_ sensitivity. Very recently, five QTLs associated with stability of SI in *B. rapa* have been identified. Two of them co-localized with *SLG* (A07) and *MLPK* (A03) and the other three were on A02, A06, and A10 (Hatakeyama *et al.*, 2010). CO_2_ sensitivity did not link with the *S*-locus in the present study (Fig. 5) and none of the other reported loci co-localized with QTLs detected here (Fig. 8), indicating that CO_2_ sensitivity of the lines in this study is determined by novel genes different from those known to affect SI stability. Genes in *BrSIO1* and *BrSIO2* regions have 103 and 243 homologues in *A. thaliana*, respectively, and 54 and 141 of these genes are expressed in *A. thaliana* pistil. *In silico* comparative analyses identified several co-expressing genes and highly homologous genes encoded in these two regions. All these can be candidate responsible genes; however, to dentify the genes responsible for high CO_2_ sensitivity in the QTL regions in *B. rapa* more accurately, it would be necessary to narrow down the regions by developing near-isogenic lines (NILs).

To maintain F_1_ seed quality, inbred lines with strong but CO_2_-sensitive SI are ideal for F_1_ hybrid breeding, and it is very important to understand the genetic relationships between SI-related genes and CO_2_ sensitivity phenotypes. These results could be useful for the marker-assisted selection of parental lines with both stable SI and high CO_2_ sensitivity.

## Supplementary data

Supplementary data are available at *JXB* online.

Figure S1. *S*-haplotype analysis of F_2_ plants by PCR-RFLP.

Figure S2. RLSICO_2_ in CO_2_-sensitive and CO_2_-insensitive lines, and F_1_ and F_2_ progeny based on the number of penetrating pollen tubes after self-pollination under high CO_2_ conditions.

Table S1. Genetic markers and their primers used for linkage analysis.

Table S2. *S*-haplotype segregation in the F_2_ population.

Table S3. Annotated genes and *Arabidopsis* homologues in *BrSIO1*.

Table S4. Annotated genes and *Arabidopsis* homologues in *BrSIO2*.

Supplementary Data

## Abbreviations:

InDel: insertion/deletion
LG: linkage group
LOD: log of odds
QTL: quantitative trait locus
RFLP: restriction fragment length polymorphism
RLSICO_2_: reaction level of SI to CO_2_
SI: self-incompatibility
SSR: simple sequence repeat.
