# Supplementary material for: Physiological and genetic analysis of CO2-induced breakdown of self-incompatibility in Brassica rapa
Source: J Exp Bot. 2013 Dec 27;65(4):939–51. doi: 10.1093/jxb/ert438 (PMC3935559; doi:10.1093/jxb/ert438)
Supplement: Supplementary Data [file supp_65_4_939__index.html]

Physiological and genetic analysis of CO2-induced breakdown of self-incompatibility in Brassica rapa — Physiological and genetic analysis of CO2-induced breakdown of self-incompatibility in Brassica rapa — Supplementary Data 

# Physiological and genetic analysis of CO2-induced breakdown of self-incompatibility in *Brassica rapa*

## Supplementary Data

Data files

**Files in this Data Supplement:**

- Supplementary Data - Supplementary Data
