# Supplementary material for: Physiological and genetic analysis of CO2-induced breakdown of self-incompatibility in Brassica rapa
Source: J Exp Bot. 2013 Dec 27;65(4):939–51. doi: 10.1093/jxb/ert438 (PMC3935559; doi:10.1093/jxb/ert438)
Supplement: Supplementary Data [file supp_ert438_jexbot106187_file001.pdf]

Table S1. *Genetic markers and their primers used for linkage analysis*

Nr	Marker	Position (CM)	LG	Forward primer (5'-3')	Rverse primer (5'-3')	Type
1	XT01-007	0.00	A01	TGTAAAACGACGGCCAGTGAGAAGAAGAGAATACCTTTGGAG	GGAGGCACATAGAAGAGTGTGACT	SSR
2	sau_um364	1.36	A01	TGTAAAACGACGGCCAGTTCTACACGACGCACTCTCTCTCT	GAGCGGTAAGGGAAGTTTGAG	SSR
3	ACMP00271	23.67	A01	TGTAAAACGACGGCCAGTCGCAGCTACTCTCATCTCCA	CGTCTCCACCAATGACCATA	SSR
4	XT01-019	33.08	A01	TGTAAAACGACGGCCAGTCTTTGGGAGTTCAGCCGATTCG	CTTTAGTACCATTAAGGTTGG	SSR
5	Ra2-G09	34.63	A01	TGTAAAACGACGGCCAGTACAGCAAGGATGTGTTGACG	GATGAGCCTCTGGTTCAAGC	SSR
6	BRMS-056	40.12	A01	TGTAAAACGACGGCCAGTGATCAAGGCTACGGAGAGAGAG	CGTGACGCTAGAGTAATCGAGT	SSR
7	BRMS-220	40.79	A01	TGTAAAACGACGGCCAGTCCTGAATGTTTTAGGAGTGTACTTC	TTGCTTCAGAAGGAAGGTCAGGAGA	SSR
8	XT01-025	45.11	A01	TGTAAAACGACGGCCAGTGACTCGGTCGCAGAGATACTGC	CCTCCATTGTCAAACCACCTTCATC	SSR
9	XT01-KS40530	51.17	A01	GGAAGGTTTGACATGGTGGAC	GTTGACAGAGTAACCCTCTGGACT	InDel
10	XT01-046	70.24	A01	TGTAAAACGACGGCCAGTGATTTGAACAATTGAAAAGCATGA	AGCAGACCCAACTTTAGGATTGTG	SSR
11	XT01-045	70.49	A01	TGTAAAACGACGGCCAGTCTATTACTTAGGGCATTTATATTGAG	CGTAATCTGAAGCATACATTTATTGG	SSR
12	BRMS-180	71.75	A01	TGTAAAACGACGGCCAGTATTAAAGTCTTTCACTCACCAGCTT	AGTCACTCTATCCGGCTGTACC	SSR
13	ACMP00756	72.35	A01	TGTAAAACGACGGCCAGTTCTCATCTTTGCCGTCTCAC	GGATTGGGTTGGTCTTCTTC	SSR
14	ACMP00617	72.82	A01	TGTAAAACGACGGCCAGTAACTCAATGCTCTTCGCTCA	CCTTCTGCTGCTTCCAAGAT	SSR
15	XT01-034	79.89	A01	GGTTTCGTTGTCCTATCGAGTCAC	AAACATCGAAAACCTCCTTCATGC	InDel
1	KBrS005A14R	0.00	A02	TGTAAAACGACGGCCAGTATTCAAATCCTTTTGGGTTCATGC	GTTTCGGTATTGGTCATACATTGTTCAAG	SSR
2	KBrB019M05R	21.05	A02	TGTAAAACGACGGCCAGTACTGCAAAGCATTGGATCTTCTCT	GTTTCCATTTCGAAGGAGAACAAAAGGA	SSR
3	Bra020149	34.85	A02	CTAATACAGGCTTTGATTTCGC	ATACCACTACTCGAGGCTAAGGAAG	RFLP
4	KBrB071M18	41.75	A02	TGTAAAACGACGGCCAGTACCGTGATTTTCTCGGTAAAGA	GTTTATCCCACAAATGTGCCTCATA	SSR
5	Bra023379	58.25	A02	CTTCGTCTTTCAAAAGAGAGGTATG	ATGAGAGAGTTAAACTGGTGTGACC	InDel
6	KBrH079A14F	87.54	A02	TGTAAAACGACGGCCAGTAGCTTTCCTACCTTTTTCCCCTTC	GTTTGAGTGCGACTTGGAATTTCTCCAT	SSR
1	BRMS-210	0.00	A03	TGTAAAACGACGGCCAGTACTTTCCTGCATGAGATATGGA	TCGGAATTGGATAAGAATTCAA	SSR
2	Ra3-D02B	29.02	A03	TGTAAAACGACGGCCAGTCACAGGAAACCGTGGCTAGA	AACCCAACCTCAACGTCTTG	SSR
3	BRMS-133	30.52	A03	TGTAAAACGACGGCCAGTAATCCCCTCTACCAACCTCATTTCT	GATCTTGCAATAAAACCGACTTTGA	SSR
4	BRMS-114	31.25	A03	TGTAAAACGACGGCCAGTGATCTCTCTTTTCCTCTTCCTTCCT	TGTTCTGTATTTTTATTTTCTCCGG	SSR
5	KBrH110I17R	50.67	A03	TGTAAAACGACGGCCAGTAGCTAAGCTAGAGCTTGCACTGAA	GTTTGCGGAACAAAGAGAAATGGAGAAA	SSR
6	BRMS-050	58.87	A03	TGTAAAACGACGGCCAGTAACTTTGCTTCCACTGATTTTT	TTGCTTAACGCTAAATCCATAT	SSR
7	BRMS-042-2	61.33	A03	TGTAAAACGACGGCCAGTAGCTCCCGACAGCAACAAAAGA	TTCGCTTCCTTTTCTGGGAATG	SSR
8	KBrH091J15R	69.71	A03	TGTAAAACGACGGCCAGTACTTCCCCGCAGATTTGAGTATAA	GTTTACGGTAACATCTTTGAGTAAGGTGA	SSR
9	BRMS-223	74.85	A03	TGTAAAACGACGGCCAGTAGCCTTGTTGCTTTTCAACG	GCTCTCAACTTGTTGTTGAGTGTTA	SSR
10	BRMS-206	77.73	A03	TGTAAAACGACGGCCAGTATTAGACTCTTTCAAAAACGCAAAG	GATGACAACAACTCTTCCTCTGTTA	SSR
11	KBrH125H23F	83.73	A03	TGTAAAACGACGGCCAGTAAAAGACAAAACCATCCAAACACTA	AAGCCTTTTTGAACTTCTCTGTGAT	SSR
12	BRMS-158	86.58	A03	TGTAAAACGACGGCCAGTACCAGTAAAACAACATCACGACCA	GTTTATGATTTACATGCCGCTGCCTC	SSR
13	XT03-003	106.96	A03	TGTAAAACGACGGCCAGTTGAGTTTTCAGATTCCGACAAC	CTCCTATAGGCCTCGTCACCAC	SSR
14	MLPK	113.65	A03	TGTAAAACGACGGCCAGTGCACAAAGCATATTTGAAGAGGATC	TAACTCCGCGTTTGGCTACAGTC	SSR
15	BRMS-043	123.69	A03	TGTAAAACGACGGCCAGTGCGATGTTTTTTCTTCAGTGTC	TTAATCCCTACCCACAATTTCC	SSR
16	sau_um034	132.65	A03	TGTAAAACGACGGCCAGTCGTTCTCTCCATCTTCACTCTCC	CTCAACTGAAGCTGCTCTTCCT	SSR
17	KBrH102J11R	151.59	A03	TGTAAAACGACGGCCAGTAGGCTTTGATCCATTCCCTTAGTG	GTTTCCCATCTCAGCAAACACAGTTACG	SSR
18	EST_0098_1343	152.55	A03	TGTAAAACGACGGCCAGTAGGCTTTGATCCATTCCCTTAGTG	GTTTCCCATCTCAGCAAACACAGTTACG	SSR
19	KBrB054N11R	160.40	A03	TGTAAAACGACGGCCAGTATCCAAACGATTTCAGATCTGCTG	GTTTGCACACACCAATTCTTCTTCG	SSR
20	KBrS016H10R	163.66	A03	TGTAAAACGACGGCCAGTACTTTGCCTGTTTGCTCTAGCGAC	GTTTGTAGCAACCACATTTGCTGCATT	SSR
21	KBrH052O08F	168.42	A03	TGTAAAACGACGGCCAGTAGCAGTAATCACTCTTGTAGCCGC	GTTTCAATCTTTTGTCCGAACCAAATCC	SSR
1	KBrH103G17F	0.00	A04	TGTAAAACGACGGCCAGTACAGACAAAAGCTTCATTGCCACA	GTTTGAGACAAAGGCTGATTCCTCCAAA	SSR
2	BRE-1_C08	10.39	A04	TGTAAAACGACGGCCAGTAGAAATCTATTTTCTTCATCTTCCA	GTTTCATCACCCAAATCGAAAG	SSR
3	ARC1	16.31	A04	CACCATGGCCACTGATTCAGCAATGT	GATCACAGGATCGTTCATGAGGTTG	RFLP
4	sau_um167	24.76	A04	TGTAAAACGACGGCCAGTGGGGAAGGAGAGAGAGTTGC	CGATAGTTCTCCTCGATGTGCT	SSR
5	sau_um042	34.60	A04	TGTAAAACGACGGCCAGTCTCTCCATCATCTTCCTCTCCTC	ATGTTCTTGTGCGGCTTCTC	SSR
6	KBrB080B22F	47.79	A04	TGTAAAACGACGGCCAGTAGCCTTTTGCTTTTTCATCTGCTA	GTTTGGAGCCCAATGAAAGTGGTAGAAA	SSR
7	BRMS-276	56.39	A04	TGTAAAACGACGGCCAGTGACCGTTTTGCATTTTAAGAGCATT	TCACCACCACCAGTATCTTCAACAATCA	SSR
8	Ol10-C01	67.05	A04	TGTAAAACGACGGCCAGTATGACTGCTTAAACAGCGCC	CTTCTCCAACAAAAGCTCGG	SSR
9	KBrB047M10F	79.29	A04	TGTAAAACGACGGCCAGTATGCCTTGTTCCATCCTCCATAAC	GTTTGACCATAGTAAAGGCGTTCCG	SSR
1	Bra029671	0.00	A05	CTTCTACTGGATGTGTATAGTCCG	AGCCTATCATTACTCGGAGAGTTC	RFLP
2	sau_um014	11.66	A05	TGTAAAACGACGGCCAGTGAATCTGGTTCCGGTTCAGTCT	CTCCTCATCCACTCCTTCTTCTC	SSR
3	XT05-001	28.65	A05	AGAGTGACTCCTCTCTTTAGCCAAC	CTGATCGTCATCACCTCTATCG	RFLP
4	BRE-1_F07	31.14	A05	TGTAAAACGACGGCCAGTATTATCAAACAGCCAACCAAAA	GTTTAGAGACTGACGGAACCTTTGAA	SSR
5	sau_um419	37.44	A05	TGTAAAACGACGGCCAGTCGCTGAAAAAGGTGGTACTCGT	GGAGCCACGGTTATGAGGATTAG	SSR
6	sau_um366	41.15	A05	TGTAAAACGACGGCCAGTTTCTCCTCGTATCACCACTCCT	GCCTACGTCTTCTACAGCGAGAT	SSR
7	KBrH070F21R	44.43	A05	TGTAAAACGACGGCCAGTAGTTCCAAACATCTGAAACCAGTG	GTTTACTCAAGCTGCGTCCAAAAGC	SSR
8	sau_um062	49.21	A05	TGTAAAACGACGGCCAGTGAACAGCAGCAGGAGGAGAC	CGAACATGGACCAAGAGAGAAC	SSR
9	KBrH059H18R	58.87	A05	TGTAAAACGACGGCCAGTACCCACCTTTTCTAGATCTGTATGA	GTTTCTCCAGCTCTTCCATCTGTTTCC	SSR
10	KBrH088D21F	64.62	A05	TGTAAAACGACGGCCAGTACTTGTGACGGAGGCATAGACTTT	GTTTGTCCCTGGAATGTCAAAGGTT	SSR
11	KBrH061C03F	73.01	A05	TGTAAAACGACGGCCAGTATACCTGCTGCTCTTGTTCCTGTA	GTTTGAGATGGCTGATGCTGAAGAAC	SSR
12	KBrB075I01F	75.08	A05	TGTAAAACGACGGCCAGTACCGTGATTTTCTCGGTAAAGA	GTTTATCCCACAAATGTGCCTCATA	SSR
13	XT05-004	78.50	A05	GCTTTTACCTGGAGCTTCTTCAGG	CTAACCAAGGCCACTCCATCACG	SSR
14	BRMS-034	91.28	A05	TGTAAAACGACGGCCAGTGATCAAATAACGAACGGAGAGA	GAGCCAAGAAAGGACCTAAGAT	SSR

Table S1. (*continued*)

Nr	Marker	Position (CM)	LG	Forward primer (5'-3')	Rverse primer (5'-3')	Type
1	Na12-D04	0.00	A06	TGTAAAACGACGGCCAGTACGGAGTGATGATGGGTCTC	CCTCAATGAAACTGAAATATGTGTG	SSR
2	BRMS-077	19.08	A06	TGTAAAACGACGGCCAGTCCGCCGAATTTATAGTGTTTTTACC	TCGATTAACCAGCTAAAATAATAATG	SSR
3	Ra2-F04	25.99	A06	TGTAAAACGACGGCCAGTCCTACAAACACATAAATAAAGAGAGAG	AACAACATAAAAGATTCATTTCG	SSR
4	BRMS-309	45.76	A06	TGTAAAACGACGGCCAGTCAAGAGCAAGTTTGAAACAAACGAT	CATCAGTTCTTGATATGCTAGGTGA	SSR
5	ACMP00692	52.69	A06	TGTAAAACGACGGCCAGTCGAGTTGCAGAGCCAAGTAG	AACGTAACGCTTCCTCTTCC	SSR
6	BRMS-221	54.39	A06	TGTAAAACGACGGCCAGTAAAGTCCTTGACGTTTGAGGAAGAA	CAGGTTCTTATGAAGGACCATGCAT	SSR
7	BRMS-095	61.95	A06	TGTAAAACGACGGCCAGTGTCATAACCAATGTATGTGTCTGCT	GATCCCTAATCAAAGAGACAGAGAG	SSR
8	ACMP00520	63.48	A06	TGTAAAACGACGGCCAGTCGGGCTTTCTAGTTGGGTAA	CGAGGTCTTACTCGATGCAG	SSR
9	KBrH101E14	72.78	A06	TGTAAAACGACGGCCAGTATTCGAAATCAAATCAAACCGCTC	GTTTCATCCAACCACGTTTTCACAGA	SSR
10	KBrH107H15R	82.90	A06	TGTAAAACGACGGCCAGTACAGCGAGTGGTAGTGACACGAAA	GTTTGCCTATCGTTCTCCTCATCTGCAT	SSR
11	EST_0098_1347	85.71	A06	TGTAAAACGACGGCCAGTATCTCTCTCTCCGAATCCAAAA	GTTTAGCTCTGATGAGACCAGCTCTT	SSR
12	Bra026007	95.57	A06	GTGGTAATGAGAGTTGGGTGTTTC	TTAGATAGAACTGGCTCCCTGAAC	InDel
13	Ol10-D01	108.66	A06	TGTAAAACGACGGCCAGTTCTCTGCCAAAAGCAAATAGC	CTTGGCTCTCTCTCACCACC	SSR
1	KBrH001H12F	0.00	A07	TGTAAAACGACGGCCAGTAGGTGAGTGCTCAGGTTCAGGTAG	GTTTCTCTGTTGCCATTGATTCGGTAA	SSR
2	Ni4-B03	8.37	A07	TGTAAAACGACGGCCAGTTGCTGTTTCTGTGGAATTGTCG	ACTTTCTTTACATTCTAATCGC	SSR
3	KBrH125E20R	13.76	A07	TGTAAAACGACGGCCAGTACATTCACTTGTAGTCCGCTCAGA	GTTTAAATGTTCGAACTGAGGTTTGGGA	SSR
4	BRMS-296	16.30	A07	TGTAAAACGACGGCCAGTCATCCTAATGTTGCTGAGAAAGAGG	TATATGAAACCGATGAAGCTCCTTT	SSR
5	KBrH107O12F	25.34	A07	TGTAAAACGACGGCCAGTACAATGCTGGGAAGAAACTCTGTG	GTTTAGATCTCTCCTCTCGCACCTCAAA	SSR
6	KBrH106E08F	29.28	A07	TGTAAAACGACGGCCAGTATCGATCCTTTGGATCTGCTTGAT	GTTTAACCGCTCCAAAAGCGTTTAATTC	SSR
7	KBrS011B13F	35.78	A07	TGTAAAACGACGGCCAGTAGACCAGCTGCAAATGAGGTTAGA	GTTTAAAAGGGTATACAGGGGGTGCAGT	SSR
8	SLG	49.36	A07	ATGAAAGGCGTAAGAAAAACCTA	CCGTGTTTTATTTTAAGAGAAAGAGCT	RFLP
9	BRMS-298	59.47	A07	TGTAAAACGACGGCCAGTCCACTGTTTTATGACTCCAGTGCTT	TGACCTGGTGAAGTAGTTGTCTCGT	SSR
10	KBrB056L15	76.09	A07	TGTAAAACGACGGCCAGTAGGACATCTTTTTGTAACCCAA	GTTTCACCTCAATTCCTAATTTTGAGA	SSR
1	BRMS-033	0.00	A08	TGTAAAACGACGGCCAGTGCGGAAACGAACACTCCTCCCATGT	CCTCCTTGTGCTTTCCCTGGAGACG	SSR
2	XT08-028	13.50	A08	GACTCCTTGTCTCCTTCTTCCTC	TGACTCCGTGAAATATAGGATCG	SSR
3	ACMP00373	18.08	A08	TGTAAAACGACGGCCAGTGCAAGTTGGGAGATTTGGAT	GCCTCAAAGGTCATCATCAG	SSR
4	Ra2-E12	25.31	A08	TGTAAAACGACGGCCAGTTGTCAGTGTGTCCACTTCGC	AAGAGAAACCCAATAAAGTAGAACC	SSR
5	Na12-B05	42.85	A08	TGTAAAACGACGGCCAGTCAAATATCCGTCATCGGAGC	CCTGCGGGATATTGAAGACC	SSR
6	KBrH077O23F	62.31	A08	TGTAAAACGACGGCCAGTACCTGTAATGTGACCCCCAACAAT	GTTTAGTCAAATGGCTCTCTCGCCAA	SSR
7	sau_um297	69.64	A08	TGTAAAACGACGGCCAGTGGAAACACGAGTTGTCACACTTA	ATGGTGAGATTGTGAAGAGGATG	SSR
8	sau_um306	87.00	A08	TGTAAAACGACGGCCAGTGTCTACTTGGTGGTTGTTAGCCC	GTGTCCTCTTCTTCAGGTTCCTC	SSR
9	EJU3	88.21	A08	TGTAAAACGACGGCCAGTACCTCTTTTAATTCAAACAAGAAATCA	GTTTTCGGACAATGGCAGTGATA	SSR
1	KBrB016E19R	0.00	A09	TGTAAAACGACGGCCAGTATGTTGAGCTGCGAATACGTTCCT	GTTTGAGCAATCTTCACCCTCTTCGTGT	SSR
2	sau_um105	1.05	A09	TGTAAAACGACGGCCAGTCCTTTCTAATGGGAAGCGGTAG	CTCCCTCTTCGAATTGACTCAC	SSR
3	Bra026855	23.56	A09	GAGTGGTGTCTGAGATCATGAGG	CTAGAAGGAAGAGAGACCAGCAAG	InDel
4	ENA21	31.50	A09	TGTAAAACGACGGCCAGTAGAGTGTTTGGAGCAGATGA	GTTGGAGACTTTGCCTTTGTGT	SSR
5	Na10-B07b	44.15	A09	TGTAAAACGACGGCCAGTGCCTTAGATTAGATGGTCGCC	ACTTCAGCTCCGATTTGCC	SSR
6	KBrH143P19R	50.88	A09	TGTAAAACGACGGCCAGTATAGACTCTTCCACCAAGTGGCAT	GTTTCGCATCCCAAATCTTGTTGAAT	SSR
7	KBrH120K23R	56.21	A09	TGTAAAACGACGGCCAGTAGAATTTTGGGGTCTGTACCTGCT	GTTTAACAGACTGGGCGTTACGGAAA	SSR
8	BRMS-079	60.26	A09	TGTAAAACGACGGCCAGTGGGAAGATCCCAATCTAAGAAAATG	AATGACCTTTGTGACCCTATTCGTT	SSR
9	Bra036837	66.33	A09	TGTAAAACGACGGCCAGTTACTGACGTGACGTATACAGATGC	CTCCTTCACGTTTTCATCATTAACC	SSR
10	Ra2-A11	80.64	A09	TGTAAAACGACGGCCAGTGACCTATTTTAATATGCTGTTTTACG	ACCTCACCGGAGAGAAATCC	SSR
11	BRMS-142	84.52	A09	TGTAAAACGACGGCCAGTTACTTTGCTTTATTTGCATGGAAGG	TTGTTTGCATTGCATGTAAGACTTC	SSR
12	BRMS-312	86.81	A09	TGTAAAACGACGGCCAGTCGCAGCAATTAAAAGCTTCACAGTA	ATTTCAACATGTCAGACGCTCTTTC	SSR
13	BRMS-120	93.16	A09	TGTAAAACGACGGCCAGTGTCAGCATACACGGCAAAACTCGCA	GGTACGGCAGTTCTCGGTCGGTTAA	SSR
14	BRMS-154	98.45	A09	TGTAAAACGACGGCCAGTATGTAGACCGAAGAAAACAAAATCA	GGAACTTTCCAGACTTGTACTCCTC	SSR
15	KBrB060H21R	104.91	A09	TGTAAAACGACGGCCAGTATGCACGAGGTTACATTATTGTGTCT	GTTTGTAAGGCCGTGTGAACTCTCT	SSR
1	KBrH080A08(BrFLC1)	0.00	A10	TGTAAAACGACGGCCAGTATGAACCTATTTTAGATTATAGCTGGC	GTTTGATGTTTTCTTCTTCTTTTTCATC	SSR
2	KBrB053N06F	9.46	A10	TGTAAAACGACGGCCAGTAGATCTCCCATGGCTTCTTGATGT	GTTTCCATTATAAGGCCCACGAAACAAA	SSR
3	KBrH094B16F	31.28	A10	TGTAAAACGACGGCCAGTACTTCTTCTCGATCTCACTCTCGG	GTTTCGAACTCCTCCTTGAACGACTC	SSR
4	KBrH006K06R	36.53	A10	TGTAAAACGACGGCCAGTAAGAGAGAGAGACGACACACACTCGG	GTTTAATCGCAATCCCCAAAAGAGAGA	SSR
5	KBrH054N12R	43.19	A10	TGTAAAACGACGGCCAGTACCATGGTAACATCTGGAAGGTGA	GTTTGTGCAGGCACTTCTACACCAG	SSR
6	sau_um217	49.81	A10	TGTAAAACGACGGCCAGTAACCAGAAACGGTGTCATGC	GTACTCCGGAAGCTCACTAAACG	SSR
7	BRMS-197	57.52	A10	TGTAAAACGACGGCCAGTCTATTACAAGTGCACTAACCCGAAT	GCTCTCAACTTGTTGTTGAGTGTTA	SSR
8	sau_um433	58.30	A10	TGTAAAACGACGGCCAGTAAGAGTCCACAGCAGGAGATTG	GGGATGAGAAAAAGACAGGTGG	SSR
9	BRMS-239	59.95	A10	TGTAAAACGACGGCCAGTAATATTCGTCTGATTATTCGGATTC	TCTCTCTGATGACTCCATACTTTCC	SSR
10	KBrB069K03F	61.12	A10	TGTAAAACGACGGCCAGTAGCATTATTTTGCACAAGAGACGG	GTTTGGGTTGCGTTGTGTAATCC	SSR
11	KBrB009O19R	63.23	A10	TGTAAAACGACGGCCAGTACCCCTGGTCTTCTTAGGTCTTCC	GTTTGAAGCCTGTTGAGGTCTCTC	SSR

Table S2. *S-haplotype segregation in F_2 population*

***S*-genotype**	**$S_{46}S_{46}$**	**$S_{46}S_{55}$**	**$S_{55}S_{55}$**	**Total**	**Goodness of fit**		
**Observed**	22	64	24	110	Ratio	χ^2	*p*
**Calculated**	27.50	55.00	27.50	110.00	1:2:1	3.02	0.2-0.4
**Average of RLSICO$_2$**	2.22	2.51	2.49				

Table S3. *Annotated genes and Arabidopsis homologues in BrSIO1*

Gene ID	LG	Start	End	AGI	Description	Slide A	Slide B	Slide C	AVE
Bra004778	A05	1802120	1804421	AT2G43710	Encodes a stearoyl-ACP desaturase	355.3	361.7	372.9	363.3
Bra004779	A05	1805009	1805659	AT2G43730	Mannose-binding lectin superfamily protein				
Bra004780	A05	1806494	1807130	AT2G43730	Mannose-binding lectin superfamily protein				
Bra004781	A05	1807522	1809777	AT2G43750	Arabidopsis thaliana O-acetylserine (thiol) lyase (OAS-TL) isoform oasB	272.7	287.7	333.2	297.9
Bra004782	A05	1810520	1811050	AT2G43760	molybdopterin biosynthesis MoaE family protein	123.3	118.4	163.2	134.9
Bra004783	A05	1811154	1813258	AT2G43770	Transducin/WD40 repeat-like superfamily protein	225.5	203.2	203.4	210.7
Bra004784	A05	1814778	1816787	AT2G43790	MAP KINASE 6	207.4	162.7	178.5	182.9
Bra004785	A05	1817117	1818644	AT2G43795	unknown protein				
Bra004786	A05	1822215	1825011	AT2G43800	Actin-binding FH2 (formin homology 2) family protein	343.2	339.3	349.8	344.1
Bra004787	A05	1828516	1830271	AT2G43820	Arabidopsis thaliana salicylic acid glucosyltransferase 1				
Bra004788	A05	1831237	1832659	AT2G43820	Arabidopsis thaliana salicylic acid glucosyltransferase 1				
Bra004789	A05	1837991	1839351	AT2G43880	Pectin lyase-like superfamily protein				
Bra004790	A05	1846616	1848000	AT2G43880	Pectin lyase-like superfamily protein				
Bra004791	A05	1849377	1850884	AT2G43880	Pectin lyase-like superfamily protein				
Bra004792	A05	1851396	1853277	AT2G43910	HARMLESS TO OZONE LAYER 1 (HOL1)				
Bra004793	A05	1857759	1859109	AT2G43910	HARMLESS TO OZONE LAYER 1 (HOL1)				
Bra004794	A05	1860370	1861952	AT2G43945	unknown protein				
Bra004795	A05	1863096	1865725	AT2G43970	RNA-binding protein	145.7	150.2	139.6	145.1
Bra004796	A05	1866075	1868706	AT2G43980	inositol 1,3,4-trisphosphate 5/6-kinase 4 (ITPK4)	105.7	112.6	88.8	102.4
Bra004797	A05	1868986	1870488	AT2G43990	unknown protein	26.8	25.9	26.9	26.5
Bra004798	A05	1871248	1871856	AT2G44000	Late embryogenesis abundant (LEA) hydroxyproline-rich glycoprotein family				
Bra004799	A05	1873482	1873766	AT2G44010	unknown protein				
Bra004800	A05	1874741	1876084	AT2G44020	Mitochondrial transcription termination factor family protein	77.3	76.5	74.7	76.2
Bra004801	A05	1877612	1879360	AT2G44065	Ribosomal protein L2 family	209.7	234.3	228.9	224.3
Bra004802	A05	1881187	1889019	-	-	-	-	-	-
Bra004803	A05	1892847	1893176	AT2G44080	Encodes ARL, a gene similar to ARGOS involved in cell expansion-dependent organ growth	98.5	114.5	104.0	105.7
Bra004804	A05	1894119	1896095	AT2G44090	Ankyrin repeat family protein	73.1	74.2	96.1	81.2
Bra004805	A05	1898162	1900945	AT2G44100	GDP dissociation inhibitor involved in vesicular membrane traffic	798.2	773.1	758.3	776.6
Bra004806	A05	1902291	1903374	AT2G44120	Ribosomal protein L30/L7 family protein	1391.5	1390.2	1443.3	1408.3
Bra004807	A05	1908181	1909470	AT2G44130	Galactose oxidase/kelch repeat superfamily protein				
Bra004808	A05	1909664	1911216	AT2G44140	Peptidase family C54 protein				
Bra004809	A05	1912514	1914621	AT2G44150	Encodes a protein-lysine N-methyltransferase. Located in ER.	92.3	88.6	81.4	87.4
Bra004810	A05	1915442	1918534	AT2G44160	methylenetetrahydrofolate reductase MTHFR2 mRNA, complete				
Bra004811	A05	1919370	1921218	AT2G44190	Encodes a novel microtubule binding protein	50.6	49.0	63.4	54.3
Bra004812	A05	1921741	1923420	AT2G44195	CBF1-interacting co-repressor CIR, N-terminal;Pre-mRNA splicing factor				
Bra004813	A05	1926040	1927962	-	-	-	-	-	-
Bra004815	A05	1933683	1935558	-	-	-	-	-	-
Bra004816	A05	1936960	1939048	-	-	-	-	-	-
Bra004817	A05	1939997	1941807	AT2G44230	Plant protein of unknown function (DUF946)	96.2	86.0	85.8	89.3
Bra004818	A05	1943704	1945964	-	-	-	-	-	-
Bra004819	A05	1947849	1950090	-	-	-	-	-	-
Bra004820	A05	1952417	1954357	-	-	-	-	-	-
Bra004821	A05	1965165	1969541	-	-	-	-	-	-
Bra004822	A05	1970933	1973160	AT2G44270	Encodes ROL5, a repressor of lrx1 mutants that develop aberrant root hairs	74.2	78.7	71.0	74.6
Bra004823	A05	1974209	1976693	AT2G44280	Major facilitator superfamily protein				
Bra004824	A05	1978048	1979340	AT2G44290	Bifunctional inhibitor/lipid-transfer protein/seed storage 2S albumin superfamily protein				
Bra004825	A05	1984033	1984575	AT2G44340	VQ motif-containing protein				
Bra004826	A05	1987355	1988083	AT2G44370	Cysteine/Histidine-rich C1 domain family protein				
Bra004827	A05	1992070	1994016	-	-	-	-	-	-
Bra004828	A05	1995264	1996025	AT2G44370	Cysteine/Histidine-rich C1 domain family protein				
Bra004829	A05	1997885	1999084	AT2G44410	RING/U-box superfamily protein				
Bra004830	A05	1999513	2001278	AT2G44420	protein N-terminal asparagine amidohydrolase family protein	41.6	42.0	44.9	42.8
Bra004831	A05	2002992	2005950	AT2G44430	DNA-binding bromodomain-containing protein				
Bra004832	A05	2006506	2008503	AT2G44440	Emsy N Terminus (ENT) domain-containing protein	47.8	56.6	46.1	50.2
Bra004833	A05	2013630	2016348	AT2G44450	beta glucosidase 15 (BGLU15)	64.3	60.3	68.0	64.2
Bra004834	A05	2020029	2022302	-	-	-	-	-	-
Bra004835	A05	2025706	2029646	AT2G44450	beta glucosidase 15 (BGLU15).	64.3	60.3	68.0	64.2
Bra004836	A05	2035186	2039155	AT2G44450	beta glucosidase 15 (BGLU15				
Bra004837	A05	2042204	2066541	AT2G44460	beta glucosidase 28 (BGLU28)	22.6	19.5	18.7	20.3
Bra004838	A05	2074677	2077758	AT2G44450	beta glucosidase 15 (BGLU15)				
Bra004839	A05	2080458	2083229	AT2G44490	Encodes a glycosyl hydrolase	43.2	46.4	46.5	45.4
Bra004840	A05	2086734	2089770	AT2G44450	beta glucosidase 15 (BGLU15)				
Bra004841	A05	2099727	2101689	AT2G44500	O-fucosyltransferase family protein	185.0	191.9	183.6	186.8
Bra004842	A05	2102608	2104660	AT2G44520	cytochrome c oxidase 10 (COX10)	84.6	86.9	86.4	86.0
Bra004843	A05	2105300	2106562	AT2G44525	Protein of unknown function (DUF498/DUF598)	186.7	183.3	165.9	178.6
Bra004844	A05	2107659	2109389	AT2G44540	glycosyl hydrolase 9B9 (GH9B9)				
Bra004845	A05	2111987	2113709	AT2G44540	glycosyl hydrolase 9B9 (GH9B9)				
Bra004846	A05	2116326	2118247	AT2G44560	glycosyl hydrolase 9B11 (GH9B11)				
Bra004847	A05	2124205	2124642	AT2G44581	RING/U-box superfamily protein				
Bra004848	A05	2125608	2127401	AT2G44580	zinc ion binding				
Bra004849	A05	2129746	2130591	AT2G44600	unknown protein				
Bra004850	A05	2133155	2135330	AT2G44610	Encodes a GTP-binding protein with similarity to yeast YPT6	242.5	239.0	208.8	230.1
Bra004851	A05	2136308	2136887	AT2G44620	Encodes a member of the mitochondrial acyl carrier protein (ACP) family.	518.5	491.2	524.1	511.3
Bra004852	A05	2137601	2139679	AT2G44640	unknown protein	184.6	186.0	177.7	182.8
Bra004853	A05	2139867	2141063	AT2G44650	Encodes a chloroplast-localized chaperonin 10	686.8	690.6	647.1	674.8
Bra004854	A05	2142969	2145037	AT2G44660	ALG6, ALG8 glycosyltransferase family				
Bra004855	A05	2147596	2147974	AT2G44670	Protein of unknown function (DUF581)	479.1	485.0	473.6	479.2
Bra004856	A05	2149090	2150324	AT2G44680	Encodes casein kinase II beta chain, a CK2 regulatory subunit.	137.6	137.4	140.7	138.6
Bra004857	A05	2151542	2152819	AT2G44690	A member of ROP GTPase gene family.	34.0	40.3	29.4	34.5
Bra004858	A05	2153663	2157318	AT2G44710	RNA-binding (RRM/RBD/RNP motifs) family protein	61.7	79.6	77.6	73.0
Bra004859	A05	2158462	2159370	AT2G44730	Alcohol dehydrogenase transcription factor Myb/SANT-like family protein	56.2	48.3	45.6	50.0
Bra004860	A05	2160277	2160909	-	-	-	-	-	-
Bra004861	A05	2161320	2162135	-	-	-	-	-	-
Bra004862	A05	2162865	2164474	-	-	-	-	-	-
Bra004863	A05	2164973	2166282	AT2G44740	cyclin p4;1 (CYCP4;1)	102.9	110.4	121.4	111.6
Bra004864	A05	2169762	2171241	AT2G44745	WRKY family transcription factor	20.8	33.3	26.7	27.0
Bra004865	A05	2176019	2177933	AT2G44760	unknown protein	49.5	54.5	42.4	48.8

Table S3. (*continued*)

Bra004866	A05	2179844	2180803	AT2G44790	UCLACYANIN 2				
Bra004867	A05	2184212	2186273	AT2G44800	2-oxoglutarate (2OG) and Fe(II)-dependent oxygenase superfamily protein				
Bra004868	A05	2204575	2205768	-	-	-	-	-	-
Bra004869	A05	2207070	2208410	AT2G44810	DEFECTIVE ANTHER DEHISCENCE 1				
Bra004870	A05	2217092	2217645	-	-	-	-	-	-
**Bra004871**	**A05**	**2222111**	**2222983**	**AT2G44820**	**unknown protein**	**186.4**	**184.1**	**191.6**	**187.4**
**Bra004872**	**A05**	**2228412**	**2231516**	**AT2G44830**	**Protein kinase superfamily protein**	**137.5**	**134.7**	**137.7**	**136.6**
**Bra004873**	**A05**	**2234840**	**2235538**	**AT2G44840**	**ETHYLENE-RESPONSIVE ELEMENT BINDING FACTOR 13**	**123.2**	**116.5**	**117.9**	**119.2**
**Bra004874**	**A05**	**2237723**	**2239355**	**AT2G44910**	**ARABIDOPSIS THALIANA HOMEOBOX-LEUCINE ZIPPER PROTEIN 4**	**13.7**	**16.8**	**15.0**	**15.2**
**Bra004875**	**A05**	**2247722**	**2249159**	**AT2G44920**	**Tetratricopeptide repeat (TPR)-like superfamily protein**	**160.5**	**161.0**	**185.5**	**169.0**
Bra004876	A05	2249958	2250774	-	-	-	-	-	-
Bra004877	A05	2252356	2257044	AT2G44930	Plant protein of unknown function (DUF247)				
**Bra004878**	**A05**	**2263243**	**2264091**	**AT2G44940**	**encodes a member of the DREB subfamily A-4 of ERF/AP2 transcription factor family.**	**41.0**	**41.9**	**38.6**	**40.5**
**Bra004879**	**A05**	**2270073**	**2274586**	**AT2G44950**	**encodes one of two orthologous E3 ubiquitin ligases in Arabidopsis**	**71.2**	**78.0**	**73.0**	**74.1**
**Bra004880**	**A05**	**2275277**	**2277507**	**AT2G44970**	**alpha/beta-Hydrolases superfamily protein**	**131.9**	**125.6**	**123.2**	**126.9**
**Bra004881**	**A05**	**2278325**	**2283046**	**AT2G44980**	**SNF2 domain-containing protein / helicase domain-containing protein**	**24.5**	**28.6**	**27.1**	**26.7**
Bra004882	A05	2283365	2286657	AT2G45000	EMBRYO DEFECTIVE 2766 (EMB2766)				
**Bra004883**	**A05**	**2287086**	**2288787**	**AT2G45010**	**PLAC8 family protein**	**129.8**	**144.1**	**126.1**	**133.3**
Bra004884	A05	2290046	2294328	AT2G45030	Translation elongation factor EFG/EF2 protein				
Bra004885	A05	2296232	2297251	AT2G45040	Matrixin family protein				
Bra004886	A05	2302497	2303350	AT2G45050	Encodes a member of the GATA factor family of zinc finger transcription factors.				
**Bra004887**	**A05**	**2305445**	**2305696**	**AT2G45070**	**Sec61 Beta Subunit**	**932.4**	**956.1**	**1002.5**	**963.7**
**Bra004888**	**A05**	**2310343**	**2311104**	**AT2G45080**	**cyclin p3;1 (cycp3;1)**	**48.4**	**43.3**	**43.9**	**45.2**
Bra004889	A05	2314366	2315456	AT2G45110	member of BETA-EXPANSINS.				
Bra004890	A05	2323211	2326057	AT2G45110	member of BETA-EXPANSINS.				
Bra004891	A05	2331494	2332478	-	-	-	-	-	-
Bra004892	A05	2332987	2338977	AT2G45220	Plant invertase/pectin methylesterase inhibitor superfamily				
**Bra004893**	**A05**	**2344666**	**2347543**	**AT2G45240**	**Encodes a cytoplasmic MAP1 like methionine aminopeptidase**	**227.3**	**242.2**	**222.1**	**230.5**
Bra004894	A05	2347927	2349138	AT2G45260	Plant protein of unknown function (DUF641)				
Bra004895	A05	2349797	2352347	AT2G45270	Mitochondrial protein essential for embryo development.				
Bra004896	A05	2353963	2355351	-	-	-	-	-	-
**Bra004897**	**A05**	**2364883**	**2367127**	**AT2G45280**	**Encodes a protein similar to RAD51C**	**45.2**	**44.9**	**43.2**	**44.4**
**Bra004898**	**A05**	**2367652**	**2370453**	**AT2G45290**	**Transketolase**	**47.3**	**54.4**	**79.6**	**60.4**
Bra004899	A05	2371226	2378801	AT2G45330	RNA 2'-phosphotransferase/ transferase, transferring phosphorus-containing groups				

Genes listed in BOLD are expressing in carpel based on Microarray data NASCArrays, and signal from 3 individual slides and those average are shown.

Table S4. *Annotated genes and Arabidopsis homologues in BrSIO2*

Gene ID	LG	Start	End	AGI	Description	Slide A	Slide B	Slide C	AVE
**Bra012922**	**A03**	**21503679**	**21504755**	**AT3G49790**	**Carbohydrate-binding protein**	**37.5**	**35.7**	**42.1**	**38.4**
Bra012921	A03	21506279	21506569	-	-	-	-	-	-
**Bra012920**	**A03**	**21506932**	**21509115**	**AT3G49740**	**pentatricopeptide (PPR) repeat-containing protein**	**33.3**	**20.4**	**23.9**	**25.9**
**Bra012919**	**A03**	**21520607**	**21521649**	**AT3G49850**	**Encodes a telomeric DNA binding protein.**	**42.6**	**40.7**	**33.9**	**39.1**
Bra012918	A03	21522066	21523153	AT3G49860	A member of ARF-like GTPase family				
**Bra012917**	**A03**	**21525865**	**21527832**	**AT3G49890**	**unknown protein**	**42.3**	**35.4**	**43.1**	**40.2**
**Bra012916**	**A03**	**21528338**	**21528778**	**AT3G49910**	**Translation protein SH3-like family protein**	**2606.1**	**2733.3**	**2843.0**	**2727.4**
Bra012915	A03	21529958	21531257	AT3G49920	Encodes a voltage-dependent anion channel.				
Bra012914	A03	21533867	21534508	AT3G49930	C2H2 and C2HC zinc fingers superfamily protein				
**Bra012913**	**A03**	**21541449**	**21542267**	**AT3G49940**	**LOB domain-containing protein 38 (LBD38)**	**62.4**	**54.6**	**67.5**	**61.5**
**Bra012912**	**A03**	**21549497**	**21550945**	**AT3G49990**	**unknown protein**	**137.9**	**145.5**	**123.6**	**135.6**
Bra012911	A03	21570191	21571431	AT3G50040	unknown protein				
**Bra012910**	**A03**	**21573956**	**21574837**	**AT3G50060**	**Encodes a member of the R2R3 transcription factor gene family.**	**711.5**	**716.7**	**722.0**	**716.7**
**Bra012909**	**A03**	**21583387**	**21584617**	**AT3G50070**	**Encode CYCD3;3, a CYCD3 D-type cyclin.**	**481.9**	**467.3**	**473.2**	**474.1**
**Bra012908**	**A03**	**21588821**	**21591662**	**AT3G50110**	**Encodes a phosphatase with low in vitro tyrosine phosphatase activity**	**55.2**	**47.4**	**47.5**	**50.0**
Bra012907	A03	21592701	21594635	AT3G50120	Plant protein of unknown function (DUF247)				
Bra012906	A03	21597258	21599046	AT3G50130	Plant protein of unknown function (DUF247)				
Bra012905	A03	21600007	21602471	AT3G50140	Plant protein of unknown function (DUF247)				
Bra012904	A03	21609574	21611627	AT3G50120	Plant protein of unknown function (DUF247)				
Bra012903	A03	21620360	21621754	AT3G50390	Transducin/WD40 repeat-like superfamily protein				
Bra012902	A03	21622445	21623207	AT3G50400	GDSL-like Lipase/Acylhydrolase superfamily protein				
**Bra012901**	**A03**	**21630236**	**21630964**	**AT3G50410**	**Arabidopsis Dof protein containing a single 51-amino acid zinc finger DNA-binding domain**	**70.5**	**79.0**	**81.6**	**77.0**
Bra012900	A03	21631175	21633316	AT3G50420	Pentatricopeptide repeat (PPR) superfamily protein				
Bra012899	A03	21634500	21635632	AT3G50440	Encodes a protein shown to have methyl jasmonate esterase activity in vitro.				
Bra012898	A03	21646727	21647884	AT3G50450	Homolog of RPW8				
**Bra012897**	**A03**	**21648630**	**21650423**	**AT3G50500**	**encodes a member of SNF1-related protein kinases (SnRK2)**	**178.8**	**185.4**	**183.2**	**182.5**
**Bra012896**	**A03**	**21650998**	**21654747**	**AT3G50520**	**Phosphoglycerate mutase family protein**	**120.1**	**133.2**	**124.4**	**125.9**
**Bra012895**	**A03**	**21656123**	**21657262**	**AT3G50620**	**P-loop containing nucleoside triphosphate hydrolases superfamily protein**	**68.7**	**61.8**	**52.5**	**61.0**
**Bra012894**	**A03**	**21677192**	**21678080**	**AT3G50630**	**Kip-related protein (KRP) gene**	**93.4**	**89.3**	**80.4**	**87.7**
Bra012893	A03	21684916	21686388	AT3G50740	UGT72E1 is an UDPG:coniferyl alcohol glucosyltransferase which specifically glucosylates sinapyl- and coniferyl aldehydes.				
Bra012892	A03	21689260	21690705	AT3G50470	UGT72E1 is an UDPG:coniferyl alcohol glucosyltransferase which specifically glucosylates sinapyl- and coniferyl aldehydes.				
Bra012891	A03	21701000	21701975	AT3G50750	BES1/BZR1 homolog 1 (BEH1)				
**Bra012890**	**A03**	**21712848**	**21713864**	**AT3G50760**	**Encodes a protein with putative galacturonosyltransferase activity.**	**15.1**	**22.2**	**21.3**	**19.5**
**Bra012889**	**A03**	**21721289**	**21721900**	**AT3G50770**	**calmodulin-like 41 (CML41)**	**30.1**	**28.6**	**25.1**	**28.0**
Bra012888	A03	21729319	21731296	-	-	-	-	-	-
Bra012887	A03	21731833	21732303	AT3G50800	unknown protein				
**Bra012886**	**A03**	**21735261**	**21735878**	**AT3G50810**	**Uncharacterised protein family (UPF0497)**	**62.4**	**74.8**	**60.1**	**65.8**
**Bra012885**	**A03**	**21739425**	**21740448**	**AT3G50830**	**cold acclimation protein WCOR413-like protein beta form**	**176.7**	**172.0**	**166.3**	**171.7**
**Bra012884**	**A03**	**21743712**	**21744839**	**AT3G50860**	**Clathrin adaptor complex small chain family protein**	**84.4**	**90.3**	**87.9**	**87.6**
**Bra012883**	**A03**	**21788116**	**21789296**	**AT3G50870**	**Encodes a GATA transcriptional regulator required to position the proembryo boundary in the early embryo.**	**30.0**	**49.1**	**50.4**	**43.2**
**Bra012882**	**A03**	**21799851**	**21803215**	**AT3G50890**	**homeobox protein 28 (HB28)**	**59.6**	**52.7**	**56.7**	**56.3**
**Bra012881**	**A03**	**21806412**	**21806831**	**AT3G50900**	**unknown protein**	**12.1**	**29.1**	**14.7**	**18.6**
**Bra012880**	**A03**	**21808947**	**21810344**	**AT3G50910**	**unknown protein**	**134.8**	**141.5**	**125.2**	**133.8**
**Bra012879**	**A03**	**21810726**	**21814469**	**AT3G50920**	**Encodes a phosphatidic acid phosphatase that can be detected in chloroplast membrane fractions.**	**119.6**	**123.1**	**135.3**	**126.0**
Bra012878	A03	21819325	21820083	-	-	-	-	-	-
Bra012877	A03	21823544	21825055	AT3G50930	cytochrome BC1 synthesis (BCS1)				
**Bra012876**	**A03**	**21825545**	**21827016**	**AT3G50960**	**Encodes a protein that functions in microtubule assembly.**	**107.8**	**113.2**	**114.5**	**111.8**
**Bra012875**	**A03**	**21827741**	**21828842**	**AT3G51000**	**alpha/beta-Hydrolases superfamily protein**	**74.0**	**67.9**	**87.4**	**76.4**
**Bra012874**	**A03**	**21829127**	**21830246**	**AT3G51010**	**unknown protein**	**361.5**	**373.1**	**367.8**	**367.5**
Bra012873	A03	21830934	21831338	AT3G51020	unknown protein				
**Bra012872**	**A03**	**21833447**	**21834370**	**AT3G51030**	**encodes a cytosolic thioredoxin**	**108.0**	**118.5**	**120.6**	**115.7**
**Bra012871**	**A03**	**21835581**	**21841120**	**AT3G51050**	**FG-GAP repeat-containing protein**	**224.6**	**241.3**	**233.2**	**233.0**
**Bra012870**	**A03**	**21842601**	**21844033**	**AT3G51090**	**Protein of unknown function (DUF1640)**	**47.3**	**38.6**	**41.7**	**42.5**
**Bra012869**	**A03**	**21844794**	**21846222**	**AT3G51100**	**unknown protein**	**174.9**	**164.3**	**172.6**	**170.6**
**Bra012868**	**A03**	**21848187**	**21853892**	**AT3G51120**	**DNA binding;zinc ion binding;nucleic acid binding;nucleic acid binding**	**105.9**	**101.4**	**99.0**	**102.1**
**Bra012867**	**A03**	**21860025**	**21864871**	**AT3G51130**	**unknown protein**	**194.6**	**215.8**	**198.9**	**203.1**
**Bra012866**	**A03**	**21867468**	**21874187**	**AT3G51150**	**ATP binding microtubule motor family protein**	**70.3**	**73.0**	**68.9**	**70.7**
**Bra012865**	**A03**	**21874550**	**21875635**	**AT3G51160**	**Catalyzes the first step in the de novo synthesis of GDP-L-fucose.**	**143.6**	**139.1**	**144.0**	**142.2**
Bra012864	A03	21879896	21880840	AT3G51190	Ribosomal protein L2 family				
Bra012863	A03	21882120	21882813	AT3G51220	Plant protein of unknown function (DUF827)				
**Bra012862**	**A03**	**21884085**	**21885545**	**AT3G51240**	**Encodes flavanone 3-hydroxylase that is coordinately expressed with chalcone synthase and chalcone isomerases.**	**821.2**	**840.4**	**864.6**	**842.1**
**Bra012861**	**A03**	**21887206**	**21888537**	**AT3G51260**	**20S proteosomal alpha subunits. Interacts with SnRK, SKP1/ASK1 during proteasomal binding of an SCF ubiquitin ligase.**	**425.4**	**398.9**	**393.3**	**405.9**
**Bra012860**	**A03**	**21889549**	**21892303**	**AT3G51270**	**protein serine/threonine kinases;ATP binding;catalytics**	**55.4**	**57.8**	**59.5**	**57.6**
**Bra012859**	**A03**	**21892762**	**21894206**	**AT3G51280**	**Tetratricopeptide repeat (TPR)-like superfamily protein**	**130.2**	**136.3**	**136.0**	**134.2**
**Bra012858**	**A03**	**21895239**	**21896289**	**AT3G51300**	**Encodes a pollen-specific Rop GTPase, member of the Rho family of small GTP binding proteins that interacts with RIC3 and RIC4 to control tip growth in pollen tubes.**	**61.6**	**52.6**	**63.6**	**59.2**
Bra012857	A03	21896574	21899299	AT3G51330	Eukaryotic aspartyl protease family protein				
**Bra012856**	**A03**	**21904574**	**21904843**	**AT3G51370**	**Protein phosphatase 2C family protein**	**153.8**	**151.7**	**137.4**	**147.7**
Bra012855	A03	21907338	21907726	AT3G51380	IQ-domain 20 (IQD20)				
Bra012854	A03	21909542	21910375	AT3G51400	Arabidopsis protein of unknown function (DUF241)				
Bra012853	A03	21913022	21913759	AT3G51410	Arabidopsis protein of unknown function (DUF241)				
**Bra012852**	**A03**	**21916802**	**21917020**	**AT3G51500**	**unknown protein**	**48.2**	**50.3**	**62.4**	**53.6**
**Bra012851**	**A03**	**21917365**	**21918998**	**AT3G51520**	**diacylglycerol acyltransferase family**	**122.5**	**119.7**	**125.3**	**122.5**
**Bra012850**	**A03**	**21919767**	**21922457**	**AT3G51550**	**FERONIA**	**376.0**	**401.5**	**400.2**	**392.6**
**Bra012849**	**A03**	**21923814**	**21925904**	**AT3G51580**	**unknown protein**	**51.3**	**49.3**	**50.9**	**50.5**
Bra012848	A03	21927444	21928521	AT3G51590	Encodes a member of the lipid transfer protein family.				
**Bra012847**	**A03**	**21930765**	**21931269**	**AT3G51600**	**Predicted to encode a PR (pathogenesis-related) protein.**	**445.7**	**451.8**	**490.3**	**462.6**
**Bra012846**	**A03**	**21935886**	**21938732**	**AT3G51620**	**Tautomerase/MIF superfamily protein**	**86.5**	**92.4**	**95.1**	**91.3**
Bra012845	A03	21943489	21944494	AT3G51660	Tautomerase/MIF superfamily protein				
**Bra012843**	**A03**	**21956417**	**21961018**	**AT3G51670**	**SEC14 cytosolic factor family protein / phosphoglyceride transfer family protein**	**479.7**	**485.7**	**485.2**	**483.5**

Table S4. (*continued*)

Bra012844	A03	21958056	21958801	-	-	-	-	-	-
Bra012842	A03	21966018	21967077	AT3G51680	NAD(P)-binding Rossmann-fold superfamily protein				
Bra012841	A03	21972702	21974345	AT3G51710	D-mannose binding lectin protein with Apple-like carbohydrate-binding domain				
**Bra012840**	**A03**	**21975321**	**21976436**	**AT3G51730**	**saposin B domain-containing protein**	**572.7**	**536.3**	**516.2**	**541.7**
Bra012839	A03	21976993	21977610	AT3G51750	unknown protein				
**Bra012838**	**A03**	**21979034**	**21982030**	**AT3G51770**	**Encodes a negative regulator of 1-aminocyclopropane-1-carboxylic acid synthase5(ACS5)**	**43.9**	**44.7**	**33.4**	**40.6**
**Bra012837**	**A03**	**21987354**	**21988859**	**AT3G51780**	**A member of Arabidopsis BAG (Bcl-2-associated athanogene) proteins, plant homologs of mammalian regulators of apoptosis.**	**143.2**	**143.7**	**135.0**	**140.6**
**Bra012836**	**A03**	**21989642**	**21991826**	**AT3G51800**	**putative nuclear DNA-binding protein G2p (AtG2) mRNA,**	**910.4**	**862.2**	**1000.7**	**924.4**
**Bra012835**	**A03**	**21999149**	**22001348**	**AT3G51850**	**member of Calcium Dependent Protein Kinase**	**335.9**	**298.4**	**278.9**	**304.4**
Bra012834	A03	22012543	22013294	-	-	-	-	-	-
**Bra012833**	**A03**	**22014395**	**22021133**	**AT3G51860**	**cation exchanger 3 (CAX3)**	**189.0**	**184.3**	**180.4**	**184.6**
**Bra012832**	**A03**	**22021830**	**22022840**	**AT3G51880**	**Encodes a protein belonging to the subgroup of HMGB (high mobility group B) proteins that have a distinctive DNA-binding motif, the HMG-box domain.**	**218.6**	**213.7**	**212.6**	**215.0**
**Bra012831**	**A03**	**22023678**	**22028202**	**AT3G51895**	**Encodes a sulfate transporter.**	**363.7**	**386.5**	**370.0**	**373.4**
Bra012830	A03	22038200	22038427	-	-	-	-	-	-
**Bra012829**	**A03**	**22040320**	**22041629**	**AT3G51910**	**member of Heat Stress Transcription Factor (Hsf) family**	**143.2**	**140.4**	**129.7**	**137.8**
**Bra012828**	**A03**	**22042610**	**22048354**	**AT3G51910**	**member of Heat Stress Transcription Factor (Hsf) family**	**143.2**	**140.4**	**129.7**	**137.8**
**Bra012827**	**A03**	**22058579**	**22060504**	**AT3G51950**	**Zinc finger (CCCH-type) family protein / RNA recognition motif (RRM)-containing protein**	**667.3**	**644.2**	**633.5**	**648.3**
Bra012826	A03	22062557	22062958	AT3G52000	serine carboxypeptidase-like 36 (scpl36)				
Bra012824	A03	22064355	22067631	AT3G52030	F-box family protein with WD40/YVTN repeat doamin				
**Bra012825**	**A03**	**22066539**	**22066980**	**AT3G52040**	**unknown protein**	**294.8**	**274.3**	**316.8**	**295.3**
Bra012823	A03	22068270	22070117	-	-	-	-	-	-
Bra012822	A03	22071617	22074763	AT3G52080	encodes a cation:proton exchanger expressed in pollen				
**Bra012821**	**A03**	**22075262**	**22075790**	**AT3G52090**	**Non-catalytic subunit common to nuclear DNA-dependent RNA polymerases II, IV and V**	**560.5**	**596.4**	**607.7**	**588.2**
Bra012820	A03	22076368	22080442	AT3G52100	RING/FYVE/PHD-type zinc finger family protein				
Bra012819	A03	22081654	22082025	AT3G52130	Bifunctional inhibitor/lipid-transfer protein/seed storage 2S albumin superfamily protein				
**Bra012818**	**A03**	**22086290**	**22087251**	**AT3G52155**	**Phosphoglycerate mutase family protein**	**50.6**	**51.5**	**50.1**	**50.7**
Bra012817	A03	22087520	22089021	AT3G52160	Encodes KCS15, a member of the 3-ketoacyl-CoA synthase family involved in the biosynthesis of VLCFA (very long chain fatty acids).				
**Bra012816**	**A03**	**22089446**	**22091224**	**AT3G52170**	**DNA binding**	**106.1**	**92.3**	**79.6**	**92.7**
Bra012815	A03	22093983	22101222	AT3G52250	Encodes a protein with a putative role in mRNA splicing.				
**Bra012814**	**A03**	**22101545**	**22105804**	**AT3G52260**	**Pseudouridine synthase family protein**	**42.8**	**38.5**	**30.9**	**37.4**
Bra012813	A03	22106896	22108296	AT3G52270	Transcription initiation factor IIF, beta subunit				
Bra012812	A03	22109374	22110381	-	-	-	-	-	-
Bra012811	A03	22110751	22111510	AT3G52270	Transcription initiation factor IIF, beta subunit				
Bra012810	A03	22111762	22113450	AT3G52280	Bromodomain containing nuclear-localized protein involved in leaf development.				
**Bra012809**	**A03**	**22114190**	**22115517**	**AT3G52300**	**ATP synthase D chain, mitochondrial (ATPQ)**	**1087.3**	**1063.0**	**1107.4**	**1085.9**
Bra012808	A03	22115961	22117190	AT3G52320	F-box and associated interaction domains-containing protein				
**Bra012807**	**A03**	**22126348**	**22127526**	**AT3G52380**	**chloroplast RNA-binding protein**	**152.6**	**179.9**	**187.9**	**173.5**
**Bra012806**	**A03**	**22129315**	**22130605**	**AT3G52400**	**syntaxin protein**	**15.8**	**20.7**	**27.1**	**21.2**
Bra012805	A03	22131668	22137432	AT3G52430	Encodes a lipase-like gene that is important for salicylic acid signaling and function in resistance (R) gene-mediated and basal plant disease resistance.				
Bra012804	A03	22138538	22139263	AT3G52440	Dof-type zinc finger DNA-binding family protein				
Bra012803	A03	22146966	22148237	AT3G52450	Encodes a cytoplasmically localized U-box domain E3 ubiquitin ligase protein				
Bra012802	A03	22152538	22153290	AT3G52460	hydroxyproline-rich glycoprotein family protein				
**Bra012801**	**A03**	**22156349**	**22156975**	**AT3G52470**	**Late embryogenesis abundant (LEA) hydroxyproline-rich glycoprotein family**	**220.8**	**233.8**	**221.0**	**225.2**
**Bra012800**	**A03**	**22160012**	**22163072**	**AT3G52490**	**Double Clp-N motif-containing P-loop nucleoside triphosphate hydrolases superfamily protein**	**47.6**	**44.7**	**42.6**	**45.0**
**Bra012799**	**A03**	**22174322**	**22175740**	**AT3G52500**	**aspartyl protease family protein**	**209.0**	**230.1**	**198.8**	**212.6**
Bra012798	A03	22183180	22183584	AT4G15215	PDR13, ATPDR13; PDR13; ATP binding / ATPase/ nucleoside-triphosphatase/ nucleotide binding				
Bra012797	A03	22187168	22194238	AT4G15215	PDR13, ATPDR13; PDR13; ATP binding / ATPase/ nucleoside-triphosphatase/ nucleotide binding				
**Bra012796**	**A03**	**22195037**	**22197102**	**AT4G15240**	**Protein of unknown function (DUF604)**	**21.5**	**23.4**	**17.0**	**20.6**
Bra012795	A03	22203115	22204539	AT4G15260	UDP-Glycosyltransferase superfamily protein				
Bra012794	A03	22205236	22207291	AT4G15400	Encodes BIA1, a member of the BAHD acyltransferase family.				
Bra012793	A03	22233686	22236995	AT4G15300	a member of the cytochrome P450 gene family. molecular function unknown.				
Bra012792	A03	22241320	22244656	AT4G15300	a member of the cytochrome P450 gene family. molecular function unknown.				
**Bra012791**	**A03**	**22246023**	**22247577**	**AT4G15410**	**serine/threonine protein phosphatase 2A 55 kDa regulatory subunit B prime gamma (PUX5)**	**183.5**	**144.8**	**163.7**	**164.0**
**Bra012790**	**A03**	**22248592**	**22250134**	**AT4G15415**	**B' regulatory subunit of PP2A (AtB'gamma)**	**83.6**	**89.2**	**79.0**	**84.0**
Bra012789	A03	22254261	22255793	AT4G15417	RNAse II-like 1 (RTL1)				
**Bra012788**	**A03**	**22266555**	**22270612**	**AT4G15440**	**Encodes a hydroperoxide lyase. Also a member of the CYP74B cytochrome p450 family.**	**356.1**	**370.4**	**378.1**	**368.2**
Bra012787	A03	22270987	22271322	AT4G15460	glycine-rich protein				
**Bra012786**	**A03**	**22278433**	**22279696**	**AT4G15470**	**Bax inhibitor-1 family protein**	**689.0**	**687.9**	**695.2**	**690.7**
**Bra012785**	**A03**	**22283402**	**22285899**	**AT4G15475**	**F-box/RNI-like superfamily protein**	**131.0**	**127.0**	**124.0**	**127.3**
**Bra012784**	**A03**	**22287411**	**22288883**	**AT4G15480**	**Encodes a protein that might have sinapic acid:UDP-glucose glucosyltransferase activity**	**33.9**	**46.0**	**37.1**	**39.0**
Bra012783	A03	22290158	22293884	-	-	-	-	-	-
**Bra012782**	**A03**	**22298207**	**22299235**	**AT4G15520**	**tRNA/rRNA methyltransferase (SpoU) family protein**	**98.2**	**94.1**	**101.8**	**98.1**
Bra012781	A03	22299647	22303813	AT4G15530	Encodes a dual-targeted protein believed to act as a pyruvate, orthophosphate dikinase.				
Bra012780	A03	22306772	22309183	AT4G15550	UDP-glucose:indole-3-acetate beta-D-glucosyltransferase				
**Bra012779**	**A03**	**22312343**	**22315209**	**AT4G15560**	**Encodes a protein with 1-deoxyxylulose 5-phosphate synthase activity involved in the MEP pathway.**	**799.0**	**835.5**	**836.8**	**823.8**
Bra012778	A03	22315983	22316959	-	-	-	-	-	-
Bra012777	A03	22325280	22326834	AT4G15610	Uncharacterised protein family (UPF0497)				
Bra012776	A03	22337080	22337502	-	-	-	-	-	-
Bra012775	A03	22340095	22341323	AT4G15610	Uncharacterised protein family (UPF0497)				
Bra012774	A03	22343131	22344328	AT4G15650	unknown protein				
Bra012773	A03	22347565	22347873	AT4G15660	Thioredoxin superfamily protein				
Bra012772	A03	22351554	22353392	AT4G15720	Tetratricopeptide repeat (TPR)-like superfamily protein				
Bra012771	A03	22356457	22357317	-	-	-	-	-	-
Bra012770	A03	22357795	22359108	-	-	-	-	-	-
Bra012769	A03	22360932	22361857	AT4G15180	SET domain protein 2 (SDG2)				

Table S4. (*continued*)

Bra012768	A03	22362305	22362934	AT4G15740	Calcium-dependent lipid-binding (CaLB domain) family protein				
Bra012767	A03	22363915	22365639	AT4G15760	Encodes a protein with similarity to monooxygenases that are known to degrade salicylic acid (SA).				
Bra012766	A03	22366090	22366604	-	-	-	-	-	-
**Bra012765**	**A03**	**22368082**	**22368645**	**AT4G15770**	**RNA binding**	**353.9**	**365.7**	**342.7**	**354.1**
**Bra012764**	**A03**	**22371466**	**22371801**	**AT4G15800**	**Member of a diversely expressed predicted peptide family**	**268.7**	**228.6**	**254.2**	**250.5**
Bra012763	A03	22373442	22374391	AT4G15802	Encodes a protein with similarity to heat shock factor binding proteins. Involved in negative regulation of heat shock response. Becomes nuclear localized upon heat treatment.				
**Bra012762**	**A03**	**22377334**	**22379165**	**AT4G15820**	**unknown protein**	**26.9**	**26.1**	**32.6**	**28.5**
**Bra012761**	**A03**	**22380010**	**22383776**	**AT4G15840**	**BTB/POZ domain-containing protein**	**65.5**	**69.7**	**68.0**	**67.7**
Bra012760	A03	22385679	22386306	-	-	-	-	-	-
Bra012759	A03	22390060	22390823	-	-	-	-	-	-
**Bra012758**	**A03**	**22391541**	**22395768**	**AT4G15880**	**EARLY IN SHORT DAYS 4**	**154.0**	**158.8**	**153.0**	**155.3**
Bra012757	A03	22397979	22400200	-	-	-	-	-	-
Bra012756	A03	22403280	22404812	-	-	-	-	-	-
Bra012755	A03	22407515	22411960	AT4G15885	kinesin motor protein-related				
**Bra012754**	**A03**	**22414390**	**22419755**	**AT4G15890**	**unknown protein**	**53.7**	**41.1**	**50.5**	**48.4**
**Bra012753**	**A03**	**22425046**	**22428376**	**AT4G15900**	**Encodes a nuclear WD40 protein that is imported into the nucleus.**	**197.5**	**193.1**	**195.5**	**195.4**
**Bra012752**	**A03**	**22429268**	**22432153**	**AT4G15920**	**Nodulin MtN3 family protein**	**103.0**	**107.0**	**93.8**	**101.3**
**Bra012751**	**A03**	**22435198**	**22435729**	**AT4G15930**	**Dynein light chain type 1 family protein**	**638.5**	**609.4**	**587.3**	**611.7**
Bra012750	A03	22436076	22437330	-	-	-	-	-	-
**Bra012749**	**A03**	**22439592**	**22441476**	**AT4G16060**	**unknown protein**	**155.9**	**142.3**	**159.0**	**152.4**
Bra012748	A03	22450559	22450975	AT4G15990	unknown protein				
Bra012747	A03	22457311	22458730	AT4G15955	alpha/beta-Hydrolases superfamily protein				
Bra012746	A03	22459058	22460791	-	-	-	-	-	-
Bra012745	A03	22466885	22470221	AT4G16070	Mono-/di-acylglycerol lipase, N-terminal;Lipase, class 3				
**Bra012744**	**A03**	**22475667**	**22477136**	**AT4G16100**	**Protein of unknown function (DUF789)**	**94.4**	**92.9**	**106.9**	**98.0**
**Bra012743**	**A03**	**22487567**	**22490721**	**AT4G16110**	**ARR2; ARR2 (ARABIDOPSIS RESPONSE REGULATOR 2)**	**36.1**	**43.0**	**32.9**	**37.3**
Bra012742	A03	22492421	22493134	AT4G16141	sequence-specific DNA binding / transcription factor/ zinc ion binding				
Bra012741	A03	22494519	22496253	AT4G16143	Protein interacts with Agrobacterium proteins VirD2 and VirE2.				
**Bra012740**	**A03**	**22500143**	**22500935**	**AT4G16143**	**IMPA-2; IMPA-2 (IMPORTIN ALPHA ISOFORM 2); binding / protein transporter**	**221.7**	**212.0**	**198.1**	**210.6**
Bra012739	A03	22501816	22504975	AT4G16144	Encodes AMSH3, a deubiquitinating enzyme.				
**Bra012738**	**A03**	**22513582**	**22514946**	**AT4G16155**	**dihydrolipoyl dehydrogenases**	**162.3**	**151.2**	**133.9**	**149.1**
Bra012737	A03	22515311	22516483	AT4G16160	Homologous to pea OEP16 and barley pPORA (OEP16)				
Bra012736	A03	22517140	22518706	-	-	-	-	-	-
Bra012735	A03	22521491	22522159	-	-	-	-	-	-
**Bra012734**	**A03**	**22522902**	**22527685**	**AT4G16170**	**unknown protein**	**199.0**	**169.5**	**274.6**	**214.4**
**Bra012733**	**A03**	**22527988**	**22529356**	**AT4G16190**	**Papain family cysteine protease**	**645.4**	**620.6**	**587.7**	**617.9**
Bra012732	A03	22529512	22529985	AT4G16195	Plant self-incompatibility protein S1 family				
**Bra012731**	**A03**	**22530232**	**22531190**	**AT4G16210**	**enoyl-CoA hydratase/isomerase A (ECHIA)**	**117.9**	**125.7**	**122.7**	**122.1**
Bra012730	A03	22533528	22533827	-	-	-	-	-	-
Bra012729	A03	22536974	22537435	-	-	-	-	-	-
Bra012728	A03	22538822	22540554	AT4G16220	GDSL-like Lipase/Acylhydrolase superfamily protein				
Bra012727	A03	22543908	22545481	-	-	-	-	-	-
Bra012726	A03	22547001	22550278	-	-	-	-	-	-
**Bra012725**	**A03**	**22553701**	**22554823**	**AT4G16360**	**5'-AMP-activated protein kinase beta-2 subunit protein**	**177.7**	**164.7**	**170.6**	**171.0**
Bra012724	A03	22559834	22560680	AT4G16380	Heavy metal transport/detoxification superfamily protein				
**Bra012723**	**A03**	**22562218**	**22562475**	**AT4G16400**	**unknown protein**	**24.5**	**16.0**	**22.0**	**20.8**
Bra012722	A03	22562648	22563190	AT4G16410	unknown protein				
**Bra012721**	**A03**	**22563364**	**22565755**	**AT4G16420**	**Transcriptional co-activator.**	**172.5**	**159.9**	**179.9**	**170.8**
**Bra012720**	**A03**	**22567864**	**22569234**	**AT4G16430**	**basic helix-loop-helix (bHLH) DNA-binding superfamily protein**	**127.5**	**111.8**	**131.9**	**123.7**
**Bra012719**	**A03**	**22569490**	**22570500**	**AT4G16442**	**Uncharacterised protein family (UPF0497)**	**249.2**	**250.2**	**237.2**	**245.5**
**Bra012718**	**A03**	**22571225**	**22572303**	**AT4G16444**	**unknown protein**	**125.8**	**127.0**	**127.3**	**126.7**
**Bra012717**	**A03**	**22575731**	**22576108**	**AT4G16447**	**unknown protein**	**278.0**	**286.4**	**333.6**	**299.3**
**Bra012716**	**A03**	**22577486**	**22577899**	**AT4G16450**	**unknown protein**	**947.1**	**960.7**	**972.2**	**960.0**
Bra012715	A03	22579876	22580573	AT4G16460	unknown protein;				
**Bra012714**	**A03**	**22584641**	**22586548**	**AT4G16480**	**Encodes a high affinity H+:myo-inositol symporter.**	**57.0**	**49.2**	**59.1**	**55.1**
**Bra012713**	**A03**	**22587234**	**22588685**	**AT4G16490**	**ARM repeat superfamily protein**	**133.3**	**134.0**	**110.1**	**125.8**
**Bra012712**	**A03**	**22604200**	**22604541**	**AT4G16500**	**Cystatin/monellin superfamily protein**	**580.8**	**537.6**	**599.4**	**572.6**
**Bra012711**	**A03**	**22607186**	**22607431**	**AT4G16515**	**Encodes a root meristem growth factor (RGF).**	**102.2**	**122.4**	**119.1**	**114.5**
**Bra012710**	**A03**	**22608868**	**22609702**	**AT4G16520**	**autophagy 8f (ATG8F)**	**458.1**	**485.4**	**532.9**	**492.1**
Bra012709	A03	22615941	22616609	AT4G16530	Family of unknown function (DUF577)				
Bra012708	A03	22617874	22618839	AT4G16540	Heat shock protein HSP20/alpha crystallin family				
Bra012707	A03	22621584	22622508	AT4G16540	Heat shock protein HSP20/alpha crystallin family				
Bra012706	A03	22624845	22627299	AT4G16540	Heat shock protein HSP20/alpha crystallin family				
Bra012705	A03	22629378	22632850	AT4G16540	Heat shock protein HSP20/alpha crystallin family				
Bra012704	A03	22635782	22636458	AT4G16540	Heat shock protein HSP20/alpha crystallin family				
**Bra012703**	**A03**	**22642127**	**22650605**	**AT4G16566**	**Encodes a protein that has an unexpected bifunctional capability in vitro.**	**62.2**	**77.3**	**71.1**	**70.2**
**Bra012702**	**A03**	**22652381**	**22656889**	**AT4G16590**	**encodes a gene similar to cellulose synthase**	**55.7**	**43.2**	**51.8**	**50.2**
**Bra012701**	**A03**	**22662703**	**22663347**	**AT4G16610**	**C2H2-like zinc finger protein**	**96.5**	**117.7**	**105.8**	**106.7**
Bra012700	A03	22666240	22667907	AT4G16620	nodulin MtN21-like transporter family protein				
Bra012699	A03	22674172	22675266	AT4G16640	Matrixin family protein				
Bra012698	A03	22678821	22683532	-	-	-	-	-	-
**Bra012697**	**A03**	**22687071**	**22689558**	**AT4G16650**	**O-fucosyltransferase family protein**	**74.8**	**85.9**	**72.3**	**77.7**
**Bra012696**	**A03**	**22690909**	**22694777**	**AT4G16660**	**heat shock protein 70 (Hsp 70) family protein**	**186.7**	**177.7**	**208.6**	**191.0**
Bra012695	A03	22696243	22696489	-	-	-	-	-	-
**Bra012694**	**A03**	**22697449**	**22699659**	**AT4G16720**	**Ribosomal protein L23/L15e family protein**	**1979.6**	**2029.6**	**2110.4**	**2039.9**
**Bra012693**	**A03**	**22708404**	**22708937**	**AT4G16750**	**encodes a member of the DREB subfamily A-4 of ERF/AP2 transcription factor family.**	**15.6**	**15.5**	**17.8**	**16.3**
Bra012692	A03	22713752	22714072	-	-	-	-	-	-
Bra012691	A03	22714414	22717327	AT4G16765	2-oxoglutarate (2OG) and Fe(II)-dependent oxygenase superfamily protein				
Bra012690	A03	22722231	22723763	AT4G16820	Encodes a lipase that hydrolyzes phosphatidylcholine, glycolipids as well as triacylglycerols.				
**Bra012689**	**A03**	**22724348**	**22730873**	**AT4G16860**	**RECOGNITION OF PERONOSPORA PARASITICA 4, RPP4**	**12.7**	**12.4**	**16.4**	**13.8**
**Bra012688**	**A03**	**22731251**	**22737169**	**AT4G16860**	**RECOGNITION OF PERONOSPORA PARASITICA 4, RPP4**	**12.7**	**12.4**	**16.4**	**13.8**
Bra012687	A03	22740665	22742204	-	-	-	-	-	-
Bra012686	A03	22744793	22746772	-	-	-	-	-	-
Bra012685	A03	22748498	22749631	AT4G17200	F-box and associated interaction domains-containing protein				
Bra012684	A03	22750594	22753322	AT4G17030	Encodes EXLB1 (expansin-like B1), a member of the expansin family.				

Table S4. (*continued*)

**Bra012683**	**A03**	**22757906**	**22760123**	**AT4G17040**	**encodes the ClpR4 subunit of the chloroplast-localized Clp protease complex.**	**688.4**	**676.2**	**687.5**	**684.0**
Bra012682	A03	22760487	22762906	AT4G17050	Encodes a protein with ureidoglycine aminohydrolase activity.				
Bra012681	A03	22770001	22771524	-	-	-	-	-	-
Bra012680	A03	22775981	22781437	-	-	-	-	-	-
Bra012679	A03	22791386	22792132	-	-	-	-	-	-
Bra012678	A03	22794839	22798551	-	-	-	-	-	-
Bra012677	A03	22803983	22804240	AT4G17085	Putative membrane lipoprotein				
**Bra012676**	**A03**	**22804681**	**22806581**	**AT4G17090**	**Encodes a beta-amylase targeted to the chloroplast.**	**204.9**	**208.2**	**206.1**	**206.4**
**Bra012675**	**A03**	**22808866**	**22811066**	**AT4G17100**	**unknown protein**	**131.5**	**149.5**	**158.7**	**146.6**
Bra012674	A03	22814475	22815042	AT4G17160	RAB GTPase homolog B1A (RABB1a)				
Bra012673	A03	22826220	22826735	AT4G17215	Pollen Ole e 1 allergen and extensin family protein				
Bra012672	A03	22831432	22833660	AT4G17250	unknown protein				
**Bra012671**	**A03**	**22834749**	**22835871**	**AT4G17260**	**Lactate/malate dehydrogenase family protein**	**308.5**	**295.5**	**291.2**	**298.4**
**Bra012670**	**A03**	**22837875**	**22839995**	**AT4G17270**	**Mo25 family protein**	**146.9**	**145.6**	**147.0**	**146.5**
**Bra012669**	**A03**	**22841667**	**22843315**	**AT4G17350**	**Protein of unknown function DUF828**	**72.5**	**66.7**	**76.2**	**71.8**
**Bra012668**	**A03**	**22843726**	**22845379**	**AT4G17370**	**Oxidoreductase family protein**	**22.0**	**21.9**	**27.4**	**23.8**
**Bra012667**	**A03**	**22846092**	**22847568**	**AT4G17390**	**Ribosomal protein L23/L15e family protein**	**1970.2**	**1845.4**	**2065.0**	**1960.2**
Bra012666	A03	22850241	22851492	-	-	-	-	-	-
Bra012665	A03	22854496	22855661	-	-	-	-	-	-
Bra012664	A03	22858877	22859704	AT4G17440	Protein of unknown function (DUF1639)				
**Bra012663**	**A03**	**22870234**	**22871338**	**AT4G17460**	**Encodes a class II HD-ZIP protein that regulates meristematic activity in different tissues**	**404.1**	**411.8**	**389.1**	**401.7**
Bra012662	A03	22872072	22873607	AT4G17470	alpha/beta-Hydrolases superfamily protein				
**Bra012661**	**A03**	**22874242**	**22875107**	**AT4G17486**	**PPPDE putative thiol peptidase family protein**	**126.5**	**129.9**	**135.4**	**130.6**
**Bra012660**	**A03**	**22876088**	**22877555**	**AT4G17510**	**ubiquitin C-terminal hydrolase 3 (UCH3)**	**189.9**	**192.1**	**200.9**	**194.3**
**Bra012659**	**A03**	**22878123**	**22879891**	**AT4G17520**	**Hyaluronan / mRNA binding family**	**261.8**	**283.4**	**308.7**	**284.6**
**Bra012658**	**A03**	**22880314**	**22881657**	**AT4G17530**	**AtRabD2c encodes a Rab GTPase**	**315.9**	**309.6**	**320.7**	**315.4**
**Bra012657**	**A03**	**22886426**	**22889023**	**AT4G17550**	**Encodes a member of the phosphate starvation-induced glycerol-3-phosphate permease gene family**	**22.9**	**27.3**	**28.9**	**26.3**
**Bra012656**	**A03**	**22890900**	**22892842**	**AT4G17600**	**Encodes Lil3:1 (light-harvesting-like) protein.**	**260.8**	**278.2**	**262.2**	**267.1**
**Bra012655**	**A03**	**22897484**	**22899346**	**AT4G17615**	**Member of AtCBL (Calcineurin B-like Calcium Sensor Proteins) family.**	**94.4**	**89.8**	**95.8**	**93.3**
Bra012654	A03	22899921	22901861	AT4G17616	Pentatricopeptide repeat (PPR) superfamily protein				
**Bra012653**	**A03**	**22902680**	**22905495**	**AT4G17620**	**glycine-rich protein**	**156.9**	**176.7**	**181.3**	**171.6**
**Bra012652**	**A03**	**22906024**	**22908683**	**AT4G17640**	**Encodes casein kinase II beta (regulatory) subunit.**	**262.7**	**227.1**	**242.2**	**244.0**
**Bra012651**	**A03**	**22909790**	**22910427**	**AT4G17670**	**Protein of unknown function (DUF581)**	**396.6**	**379.8**	**388.2**	**388.2**
Bra012650	A03	22915403	22927293	AT4G17680	SBP (S-ribonuclease binding protein) family protein				
**Bra012649**	**A03**	**22933529**	**22933996**	**AT4G17690**	**Peroxidase superfamily protein**	**13.7**	**9.7**	**9.4**	**10.9**
Bra012648	A03	22947960	22948367	AT4G17718	Encodes a defensin-like (DEFL) family protein.				
**Bra012647**	**A03**	**22949396**	**22951220**	**AT4G17720**	**RNA-binding (RRM/RBD/RNP motifs) family protein**	**139.3**	**143.8**	**164.9**	**149.3**
**Bra012646**	**A03**	**22957393**	**22958909**	**AT4G17720**	**RNA-binding (RRM/RBD/RNP motifs) family protein**	**139.3**	**143.8**	**164.9**	**149.3**
**Bra012645**	**A03**	**22959734**	**22961071**	**AT4G17730**	**member of SYP2 Gene Family**	**193.5**	**195.7**	**188.8**	**192.7**
**Bra012644**	**A03**	**22961454**	**22964005**	**AT4G17740**	**Peptidase S41 family protein**	**102.7**	**112.1**	**126.6**	**113.8**
**Bra012643**	**A03**	**22965905**	**22967302**	**AT4G17760**	**damaged DNA binding;exodeoxyribonuclease IIIs**	**19.2**	**14.0**	**17.2**	**16.8**

Genes listed in BOLD are expressing in carpel based on Microarray data NASCArrays, and signal from 3 individual slides and those average are shown.

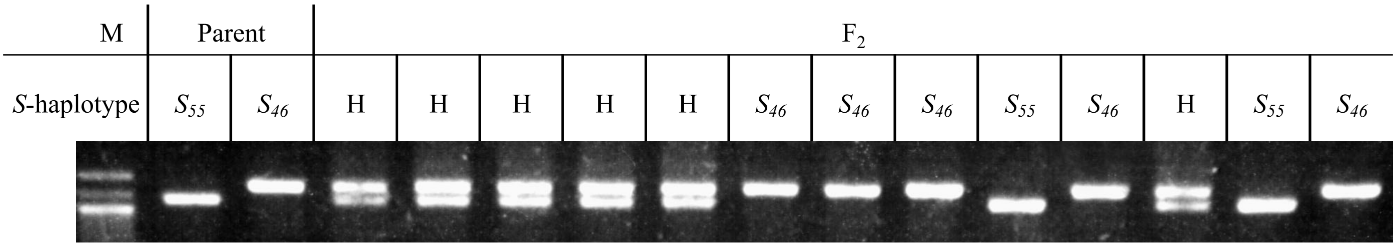

Fig. S1. *S*-haplotype analysis of F_2 plants by PCR-RFLP.
M, marker; S_{55}, $S_{55}S_{55}$-homozygote; S_{46}, $S_{46}S_{46}$-homozygote, H, $S_{46}S_{55}$-heterozygote.

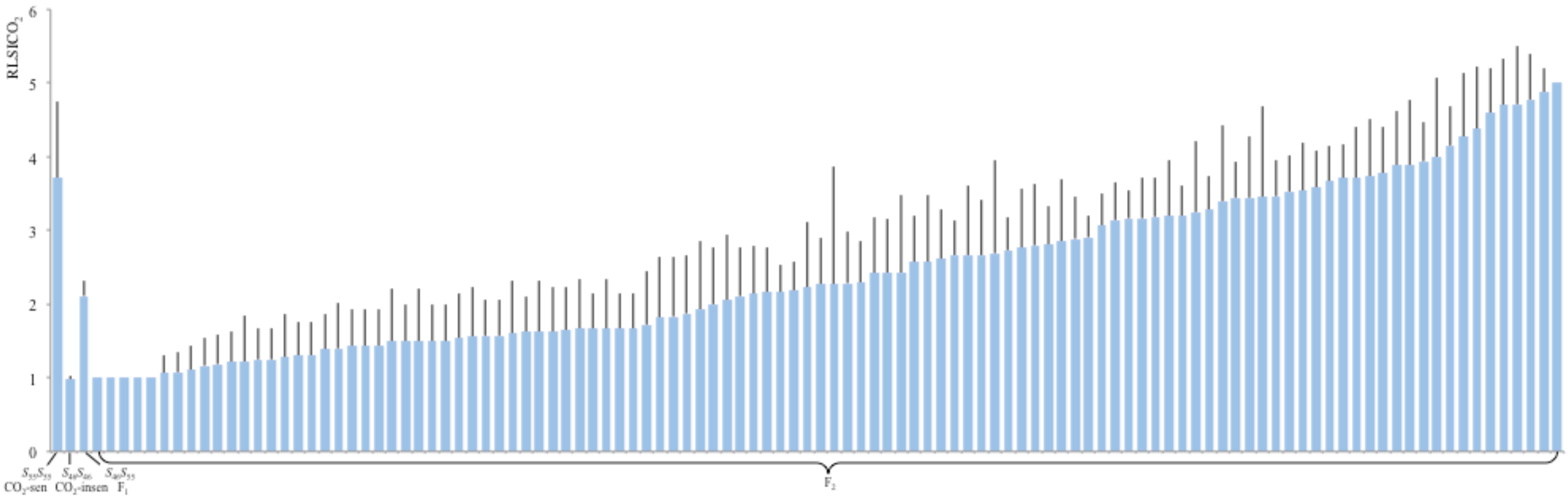

Fig. S2. $RLSICO_2$ inCO_2-sensitive, CO_2-insensitive lines, F_1 and F_2 progeny based on the number of penetrating pollen tubes after self-pollination under high CO_2 conditions. $RLSICO_2$ is continuously distributed and does not follow a simple one-locus biallelic Mendelian distribution.
